# Severe acute malnutrition promotes bacterial binding over proinflammatory cytokine secretion by circulating innate immune cells

**DOI:** 10.1126/sciadv.adh2284

**Published:** 2023-11-01

**Authors:** Tracy N. Phiri, Kuda Mutasa, Sandra Rukobo, Margaret Govha, Patience Mushayanembwa, Simutanyi Mwakamui, Tafhima Haider, Kanekwa Zyambo, Cherlynn Dumbura, Joice Tome, Thompson Runodamoto, Leah Chidamba, Florence D. Majo, Deophine Ngosa, Kanta Chandwe, Chanda Kapoma, Benjamin Mwapenya, Wadzanai Mufukari, Jonathan P. Sturgeon, Ruairi C. Robertson, Melanie Smuk, Robert Ntozini, Kusum Nathoo, Beatrice Amadi, Paul Kelly, Mutsa Bwakura-Dangarembizi, Andrew J. Prendergast, Claire D. Bourke

**Affiliations:** ^1^ Tropical Gastroenterology and Nutrition group (TROPGAN), University of Zambia School of Medicine, Lusaka, Zambia.; ^2^ Zvitambo Institute for Maternal and Child Health Research, Harare, Zimbabwe.; ^3^ Blizard Institute, Queen Mary University of London, London, UK.; ^4^ Department of Paediatrics and Child Health, University of Zimbabwe College of Health Sciences, Harare, Zimbabwe.

## Abstract

Children with severe acute malnutrition (SAM) have high infectious mortality and morbidity, implicating defects in their immune defenses. We hypothesized that circulating innate immune cells from children (0 to 59 months) hospitalized with SAM in Zambia and Zimbabwe (*n* = 141) have distinct capacity to respond to bacteria relative to adequately nourished healthy controls (*n* = 92). SAM inpatients had higher neutrophil and monocyte *Escherichia coli* binding capacity but lower monocyte activation and proinflammatory mediator secretion in response to lipopolysaccharide or heat-killed *Salmonella typhimurium* than controls. Among SAM cases, wasting severity was negatively associated with cytokine secretion, children with HIV had lower monocyte activation, and the youngest children released the least myeloperoxidase upon stimulation. Inpatient bacterial binding capacity and monocyte activation were associated with higher odds of persistent SAM at discharge, a risk factor for subsequent mortality. Thus, SAM shifts innate immune cell function, favoring bacterial containment over proinflammatory activation, which may contribute to health deficits after discharge.

## Teaser

Anti-bacterial innate immune cell function is shaped by severe acute malnutrition and associated with persistent wasting.

## INTRODUCTION

Wasting is a form of undernutrition that affects 6.7% of all children under 5 years old (45.4 million) globally, 89% of whom live in low or middle income countries (LMICs) (*1*). Severe acute malnutrition (SAM) is the most life-threatening form of wasting, clinically characterized as severe wasting [weight-for-height *z* score (WHZ) ≤ −3 and/or mid-upper arm circumference (MUAC) < 115 mm, for children aged 6 to 59 months] with or without bilateral pitting edema (*2*, *3*). Children with clinical complications of SAM requiring hospital admission [i.e., one of the Integrated Management of Childhood Illness clinical danger signs, acute infection, severe edema, or feeding problems (*2*)] are at particularly high risk of mortality; a meta-analysis of 19 studies among children under 5 years old conducted in eight African countries found a mean all-cause mortality during hospitalization of 15.7% [95% confidence interval (CI): 10.4 to 21.0] (*4*). Such high inpatient mortality rates and the persistent wasting that affects survivors of SAM compromise ambitious global development goals to reduce global mortality among children under 5 years old, reduce and maintain childhood wasting to less than 5% by 2025 (3% by 2030), and achieve zero hunger (*1*, *5*). Despite its definition according to anthropometry, it is increasingly recognized that complicated SAM is a combination of conditions rather than a single disorder (*4*, *6*–*9*) with poor clinical outcomes before and after discharge from hospital (*6*, *9*–*11*) and a pathophysiology that is not fully understood.

Pathogen carriage is common among children with complicated SAM at hospital admission (*8*, *10*, *12*–*16*), and empirical antibiotic treatment is therefore recommended even in the absence of infectious symptoms (*3*). Infections also worsen clinical outcomes among children with SAM: Infections independently predict inpatient mortality (*4*), bacteremia is a frequent complication among children who die in hospital (*16*), and the risk of infectious mortality increases incrementally with the severity of wasting (*17*). However, pathogen carriage and exposure can also be high among nonwasted and otherwise healthy children in LMIC (*18*, *19*), and children with SAM appear susceptible to a range of pathogen species and types (*8*, *10*, *12*–*16*), suggesting that poor clinical outcomes among children with SAM reflect a reduced capacity to prevent and contain infection rather than infection carriage per se. Consistent with this hypothesis, children with both wasting and stunting have higher circulating levels of pathogen-associated molecular patterns (PAMPs), including bacterial endotoxin [also termed lipopolysaccharide (LPS)] and flagellin (*18*, *20*–*23*), which are thought to derive from both disseminated infections and chronic translocation of intestinal microbes, PAMP, and microbial metabolites across their compromised gut barrier. Elevated systemic levels of proinflammatory biomarkers correspond with circulating LPS levels (*18*, *23*) and anti-PAMP immunoglobulins (e.g., anti-flagellin and anti-LPS) in undernourished children (*21*–*25*), and this inflammatory state is associated with mortality among children with complicated SAM during hospitalization and after discharge (*15*, *26*, *27*), and positively correlated with metabolic disturbances associated with inpatient mortality (*27*). Untargeted proteomic analyses of blood samples from children hospitalized with SAM in Kenya and Malawi highlight a “sepsis-like” phenotype, which is significantly associated with pre- and post-discharge mortality; the phenotype spans multiple immunoactive proteins involved in acute phase response [interleukin-6 (IL-6) (*15*) and tumor necrosis factor–α (TNFα) (*15*, *26*, *27*)], mobilization of innate immune cells [calprotectin (*26*), IL-6 (*15*), IL-7 (*27*), IL-8 (*26*, *27*), IL-15 (*26*, *27*), IP-10 (*26*), granulocyte colony-stimulating factor (G-CSF) (*15*, *27*), MCP-1 (*27*), and TNFα (*15*, *26*, *27*)], lymphocyte proliferation and survival [IL-2 (*15*), IL-7 (*27*), and TNFβ (*15*)], and vascular repair and hemostasis [von Willebrand factor (*26*) and angiotensinogen (*26*)]. However, the cellular source and mechanisms that underlie perturbations of circulating biomarkers in undernourished children are unclear. Among adults with Gram-negative sepsis, systemic circulation of bacterial PAMP not only drives functional adaptation of circulating immune cells to limit lethal systemic inflammation (*28*, *29*) but also may compromise defense against new infectious challenges (i.e., those to which patients may be exposed after their initial septic episode) (*30*–*33*). Multiple innate and adaptive immune mediators are dysregulated in undernourished children (*24*, *34*, *35*), indicative of an altered immune phenotype. However, few studies have directly assessed the capacity of immune cells to respond to new challenges (immune cell function) among cohorts of undernourished children adequately powered for their inherent clinical, demographic, and immune heterogeneity; fewer still have evaluated how clinical characteristics of complicated SAM influence immune function relative to healthy controls from the same community (*24*, *34*, *35*).

In this study, we evaluated how innate immune cells from children under 5 years old admitted to hospitals in Zambia and Zimbabwe with complicated SAM respond to bacterial PAMP challenge in vitro, a model for how each child might respond to a newly acquired infection. We focused on Gram-negative bacterial stimuli derived from species frequently identified in clinical isolates from children with complicated SAM (*Escherichia coli* and *Salmonella typhimurium*) (*10*, *14*, *15*) and the two most abundant circulating innate immune cell types, monocytes and neutrophils. We addressed three outstanding questions in nutritional immunology: (i) How does the capacity to respond to bacterial challenge differ between innate immune cells from children hospitalized with SAM and adequately nourished children? (ii) Which clinically relevant characteristics of children with SAM contribute to heterogeneity in their antibacterial innate immune cell function? (iii) Is antibacterial innate immune cell function among children with SAM associated with nutritional status at hospital discharge?

## RESULTS

### Demographic and clinical characteristics were collected on children with SAM and healthy controls

We undertook antibacterial innate immune cell function assessment using blood samples from 141 children under 5 years old who were hospitalized with complicated SAM [defined according to the World Health Organization (WHO) criteria (*2*)] and 92 children who were adequately nourished (WHZ > −1), had adverse exposures typical of LMIC {e.g., HIV infection and exposure, environmental pathogens, marginal diet, and stunting [height-for-age *z* score (HAZ) < −2]}, but were otherwise healthy with no symptoms of acute infection (i.e., community-relevant healthy controls; Table 1). All children were participants in a longitudinal observational cohort study of the health outcomes and pathogenesis of SAM [HOPE-SAM (*36*)] and were recruited from three peri-urban tertiary hospital sites in Zambia (University Teaching Hospital, Lusaka) and Zimbabwe (Harare Central and Parirenyatwa Hospitals, Harare); inclusion of HOPE-SAM participants in this study is shown in fig. S1. Although all participants in the SAM group were critically unwell, they tended to be healthier (i.e., lower mortality, slightly older, less severe wasting, and a lower percentage had persistent SAM at discharge) than those included in the wider HOPE-SAM cohort (table S1).

**Table 1.** Baseline demographic and clinical characteristics of the SAM and healthy control groups of the IMMUNO-SAM cohort. IQR, interquartile range; SD, standard deviation; ART, antiretroviral therapy; CTX, cotrimoxazole; HIV, human immunodeficiency virus; MUAC, mid-upper arm circumference; WHZ, weight-for-height *z* score; WAZ, weight-for-age *z* score; HAZ, height-for-age *z* score; CRP, C-reactive protein; sCD14, soluble cluster of differentiation 14; sCD163, soluble cluster of differentiation 163; LBP, LPS-binding protein; MPO, myeloperoxidase.

	Healthy controls	SAM inpatients
*N*	92	141
Of HOPE-SAM cohort, *n*/*N* (%)	92/174 (52.9%)	141/745 (18.9%)
Of HOPE-SAM blood samples, *n*/*N* (%)	92/173 (53.2%)	141/630 (22.4%)
Hospital site*		
Harare Central Hospital, *n*/*N* (%)	38/92 (41.3%)	54/141 (38.3%)
Parirenyatwa Hospital, *n*/*N* (%)	40/92 (43.5%)	60/141 (42.6%)
University Teaching Hospital, *n*/*N* (%)	14/92 (15.2%)	27/141 (19.1%)
Inpatient mortality^†^, *n*/*N*	–	0/141 (0%)
Time to discharge (days), median (IQR) [*n*]	–	11.0 (7, 16) [141]
Participant withdrew, *n*/*N*	–	0/141 (0%)
Discharge against medical advice, *n*/*N* (%)	–	8/132 (6.1%)
Participant characteristics (baseline)		
Age (months), median (IQR) [*n*]	26.0 (18–40) [92]	19.3 (13–22) [141]
<6^‡^	0/92 (0.0%)	7/141 (5.0%)
6–11^‡^	12/92 (13.0%)	17/141 (12.1%)
12–23	30/92 (32.6%)	92/141 (65.2%)
24–59	50/92 (54.3%)	25/141 (17.7%)
Male, *n*/*N* (%)	37/92 (40.2%)	71/141 (50.4%)
Cerebral palsy, *n*/*N* (%)	0/92 (0.0%)	7/141 (5.0%)
Birthweight (kg), mean (SD) [*n*]	3.0 (0.5) [86]	2.9 (0.6) [126]
Cessation of breastfeeding <6 months, *n*/*N* (%)	0/92 (0%)	10/141 (7.1%)
HIV status^§^		
HIV positive, *n*/*N* (%)	32/92 (34.8%)	33/141 (23.4%)
On ART, *n*/*N* (%)	32/32 (100%)	14/33 (42.4%)
On CTX, *n*/*N* (%)	26/31 (83.9%)	19/28 (67.9%)
HIV-exposed uninfected, *n*/*N* (%)	22/88 (25.0%)	19/138 (13.8%)
HIV-unexposed uninfected, *n*/*N* (%)	34/88 (38.6%)	89/138 (64.5%)
Nutritional status (baseline)		
Edematous SAM, *n*/*N* (%)	–	98/141 (69.5%)
MUAC (mm), mean (SD) [*n*]	151.6 (12.2) [90]	119.7 (18.9) [141]
WHZ, mean (SD) [*n*]	0.4 (0.9) [91]	−2.7 (1.5) [141]
WAZ, mean (SD) [*n*]	−0.6 (1.1) [92]	−3.6 (2.0) [141]
HAZ, mean (SD) [*n*]	−1.6 (1.5) [92]	−2.9 (1.5) [141]
Stunting status (HAZ < −2), *n*/*N* (%)	35/92 (38.0%)	68/141 (48.2%)
Systemic inflammatory mediators (baseline)		
Plasma CRP (mg/liter), median (IQR) [*n*]	0.2 (0.0–1.4) [92]	1.5 (0.2–8.3) [119]
Plasma sCD14 (μg/ml), median (IQR) [*n*]	1.5 (1.0–2.0) [92]	1.8 (1.3–2.2) [116]
Plasma sCD163 (pg/ml), median (IQR) [*n*]	979 (700–1289) [91]	1279 (823–2129) [117]
Plasma LBP (μg/ml), median (IQR) [*n*]	3.1 (2.0–5.0) [91]	8.6 (4.9–12.5) [116]
Intestinal inflammatory mediators (baseline)		
Stool MPO (ng/ml), median (IQR) [*n*]	1657 (390–3213) [76]	1462 (946–4933) [47]
Stool neopterin (nmol/l), median (IQR) [*n*]	373 (148–960) [79]	787 (442–2057) [33]
Clinical signs and symptoms (day of immunoassay)		
Time to first immunoassay (days), median (IQR) [*n*]	–	6.0 (4–11) [137]
Any symptom^¶^, *n*/*N* (%)	–	105/130 (80.8%)
Number of symptoms, median (IQR) [*n*]	–	2.0 (2–4) [105]
Any symptom associated with infection^#^, *n*/*N* (%)	–	78/130 (60.0%)
Number of infectious symptoms, median (IQR) [*n*]	–	2.0 (1–2) [78]

*Immune function assessments were undertaken in centralized laboratories in Zimbabwe (Harare Central and Parirenyatwa) and Zambia (University Teaching Hospital).

†Comparison of the SAM group to the wider HOPE-SAM cohort is provided in table S1.

‡Age groups according to grouping for stratification for HOPE-SAM recruitment and analysis of mortality outcomes (*9*, *36*); the <6-month and 6- to 11-month age groups were combined for analyses.

§All participants had a known HIV infection status (i.e., HIV positive or negative); four healthy controls and three SAM cases did not have data available on exposure to maternal HIV.

¶Participants were classified as having “any symptom” if the study clinician recorded “Yes” to any of the standardized list of signs/symptoms on the daily inpatient review form the day of hospitalization corresponding to the day of blood sampling.

#Clinician review recorded Yes to any sign/symptom likely to be caused by infection [i.e., persistent diarrhea, acute diarrhea, cough, sepsis, meningitis, measles in the past 3 months, malaria, tuberculosis (including suspected), skin infection, oral thrush, ear discharge, cannula site infection, urinary tract infection, and any infections in free text “other”]; HIV status and sequelae due to congenital infections were not included.

Innate immune cell function was characterized in the SAM group (Table 1) using the first available venous blood sample (≥2 ml) collected during their inpatient period [median time from admission to immune function assessment (time to immunoassay): 6.0 days, interquartile range (IQR): 4 to 11]; 38.2% of the SAM group had their first immune function assessment on the day of discharge from hospital. Clinical symptoms were recorded daily during hospitalization, with 80.8% of children in the SAM group having ongoing clinical complications typical of the complicated SAM phenotype (Table 1; prevalence of individual symptoms is detailed in table S2). Sixty percent of participants were classified as having one or more symptoms indicative of acute infection on the day that their blood sample was collected for immune function assessment; infectious symptoms recorded in the SAM group included sepsis (*n* = 1), suspected tuberculosis (*n* = 18), diarrhea (*n* = 43), cough (*n* = 34), oral thrush (*n* = 15), conjunctivitis (*n* = 1), and/or cannula site infection (*n* = 2) (table S2). All children who had immune function assessment during hospitalization survived to hospital discharge, spending a median of 11.0 (IQR: 7.0 to 16.0) days in hospital; our cohort therefore characterizes immune function as a primary outcome among children who survive inpatient treatment for complicated SAM but for whom vulnerability to mortality and nutritional and infectious morbidity persist after discharge (*6*, *9*, *37*). Healthy controls had a venous blood sample collected for immune function assessment at enrolment.

Thirty-three children in the SAM group (23.4%) and 32 children in the healthy control group (34.8%) were living with HIV. In total, 42.4% of children with HIV in the SAM group were on antiretroviral therapy (ART) at hospital admission and 100% of children with HIV children in the healthy control group were on ART. Among HIV-negative children with available data on exposure to maternal HIV infection, 19 children in the SAM group (13.8%) and 22 healthy controls (25.0%) were HIV-exposed, uninfected (HEU).

Consistent with previous studies (*24*, *34*, *35*), children with complicated SAM had higher concentrations of systemic and intestinal inflammatory biomarkers relative to healthy controls (Table 1); these included elevated monocyte/macrophage-derived surface marker levels (sCD14 and sCD163), acute phase proteins [C-reactive protein (CRP)], and LPS-binding protein (LBP) in plasma and innate immune cell granules in stool [myeloperoxidase (MPO) and neopterin].

### SAM enhances the capacity of innate immune cells to bind bacteria

To quantify the capacity of blood immune cells to contain bacteria, we incubated whole-blood samples from each child either without stimulus (negative control) or with *E. coli*–coated bioparticles labeled with a fluorescent dye [Alexa Fluor 488 (AF488)] at 37°C for 1 hour and then washed, fixed, and analyzed proportions and phenotype of *E. coli^+^* cells and their mean fluorescence intensity for *E. coli* (meanFI) via flow cytometry (fig. S2). This assay identifies cells that have bound to the cell surface and/or internalized the bioparticles and therefore reflects both bacterial binding and phagocytic capacity. The SAM and healthy control groups had similar total leukocyte counts (fig. S3A and table S3) and percentages of lymphocytes [positive for any lymphocyte lineage marker (Lin): CD3 (T cells), CD19, CD20 (B cells), and/or CD56 (natural killer cells)] and neutrophils (Lin^−^CD66b^+^CD16^+^) in unstimulated cultures (fig. S3B and table S3). Percentages of monocytes (Lin^−^CD66b^−^HLA-DR^+^) were slightly lower in unstimulated cultures from the SAM group (median: 2.3%; IQR: 1.3 to 4.0) versus healthy control group (3.7%, IQR: 2.7 to 5.3; *P* = 0.004) with lower percentages of classical monocytes [CD14^hi^CD16^−^; median: 30.6% (IQR: 16.2 to 41.0) versus 35.5% (IQR: 26.4 to 46.4); *P* = 0.025], higher percentages of intermediate monocytes [CD14^hi^CD16^+^; median: 12.0% (IQR: 7.8 to 16.0) versus 8.3% (IQR: 5.9 to 11.9); *P* < 0.001], and similar percentages of nonclassical monocytes [CD14^lo^CD16^+^; median: 29.8% (IQR: 18.1 to 40.1) versus 32.7% (IQR: 22.7 to 41.2); *P* = 0.410; fig. S3C and table S3]. The classical, intermediate, and nonclassical monocyte subsets reflect a maturation continuum (*38*); expanded intermediate monocytes therefore reflect increased circulating monocyte activation and maturation in SAM versus healthy controls. Unstimulated cultures from both groups had negligible *E. coli*^+^ events and AF488 meanFIs (fig. S2B and table S4).

**Fig. 1.**
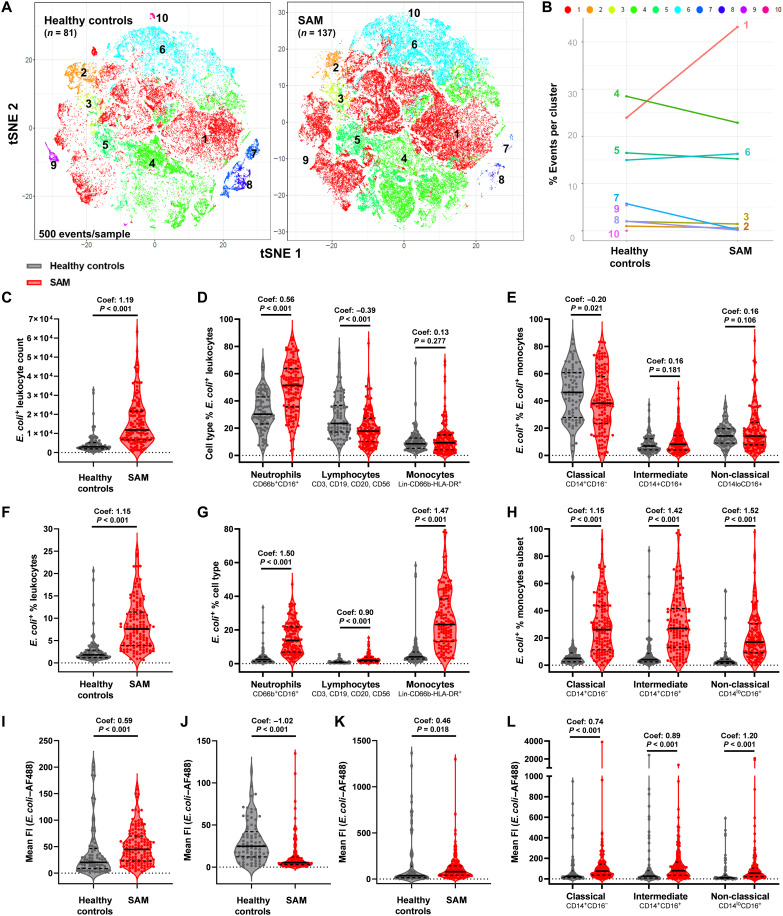
Circulating neutrophils and monocytes from children with SAM have enhanced bacterial binding capacity compared to healthy controls. (**A**) tSNE plot of 500 *E. coli*^+^ single leukocytes per sample clustered by cell surface marker expression (Lin, CD66b, CD14, CD16, and HLA-DR) and color-coded by FlowSOM clusters 1 to 10 (more detail in fig. S4) for the healthy control (left; *n* = 81) and SAM groups (right; *n* = 137). (**B**) Percentage distribution of FlowSOM clusters for *E. coli*^+^ leukocytes from healthy controls versus SAM cases. (**C**) Total *E. coli*^+^ single leukocyte count, (**D**) leukocyte subtypes as a percentage of total *E. coli*^+^ leukocytes, and (**E**) monocyte subsets as a percentage of total *E. coli*^+^ monocytes by healthy control (gray) versus SAM group (red) from hierarchical gating analysis. *E. coli*^+^ percentage of (**F**) total single leukocytes, (**G**) leukocyte subtypes, and (**H**) monocyte subsets. *E. coli* mean AF488 fluorescence intensity (meanFI) for (**I**) neutrophils, (**J**) mixed lymphocytes, (**K**) total monocytes, and (**L**) monocyte subsets. Violin plots indicate median (dark line) and IQR (dashed lines); coefficients (Coef.) and *P* values are reported for unadjusted univariable negative binomial regression of event counts (C), fractional regression of proportions (E and D to H), and linear regression of meanFI (I to L) by SAM status.

After incubation with *E. coli* bioparticles, we evaluated standardized numbers of *E. coli*^+^ leukocytes from each participant (500 events per participant; fig. S4A) via *t*-distributed stochastic neighbor embedding (tSNE) and clustering of *E. coli*^+^ cell types according to their cell surface marker expression via flow cytometry self-organizing maps (FlowSOM; Fig. 1A). These analyses identified 10 clusters of *E. coli*^+^ leukocytes across both groups (fig. S4B) with varying intensity of *E. coli* binding (fig. S4C): Clusters 3, 4, 5, 7, 9, and 10 had the highest relative expression of lymphocyte lineage markers; clusters 1 and 2 had the highest relative expression of the granulocyte marker CD66b, and cluster 1 also has the greatest labeling for CD16, which is coexpressed with CD66b on neutrophils; clusters 2, 6, and 8 had the highest relative expression of CD14, which is expressed on monocytes, and mixed expression of CD16, which is up-regulated by classical monocytes as they mature into the intermediate and nonclassical subsets (fig. S4D). HLA-DR was widely expressed across clusters, consistent with expression across innate and adaptive leukocytes (fig. S4). The SAM group had a higher percentage of events in cluster 1 (neutrophil phenotype) and cluster 6 (monocyte phenotype) and a lower percentage of events in clusters 4, 5, 7, 9, and 10 (mixed lymphocyte phenotype) than the healthy control group (Fig. 1B), indicating that the immune cell types responsible for bacterial binding differ according to nutritional status. The SAM group also had higher overall *E. coli*^+^ leukocyte counts than healthy controls (Fig. 1C; coefficients and *P* values from unadjusted models are indicated, and full details of analyses are in table S4). Consistent with unsupervised tSNE analysis, hierarchical gating of *E. coli*^+^ leukocytes demonstrated that neutrophils made up a higher percentage, lymphocytes made up a lower percentage, and monocytes made up a similar percentage of *E. coli*^+^ leukocytes in the SAM versus healthy control group (Fig. 1D; flow cytometry gating strategy in fig. S2). Within the monocyte population, classical monocytes made up a lower percentage of *E. coli*^+^ leukocytes in the SAM versus healthy control group (Fig. 1E).

To evaluate the cell type–specific capacity for *E. coli* binding, independent of compositional differences in the cell types present in the unstimulated blood sample, we compared *E. coli*^+^ percentages of total leukocytes, leukocyte subtypes, and monocyte subsets between groups. A higher percentage of total leukocytes (Fig. 1F), neutrophils, lymphocytes, monocytes (Fig. 1G), and all three monocyte subsets (Fig. 1H) bound to *E. coli* in the SAM group compared to the healthy control group, indicative of a generalized enhancement of bacterial binding capacity in the context of SAM, which was more pronounced in neutrophils (fractional regression coefficient: 1.50, 95% CI: 1.19 to 1.81; *P* < 0.001) and monocytes (coefficient: 1.47, 95% CI: 1.15 to 1.79; *P* < 0.001) than in lymphocytes (coefficient: 0.90, 95% CI: 0.65 to 1.15; *P* < 0.001).

We assessed *E. coli* meanFI as a measure of the amount of bioparticles bound per cell; neutrophils from the SAM group had higher meanFI (Fig. 1I), lymphocytes had lower meanFI (Fig. 1J), and total monocytes (Fig. 1K) and all monocyte subsets had higher meanFI (Fig. 1L) than those from the healthy control group. Thus, circulating innate immune cell types from children with SAM bind more bacteria per cell than those from healthy controls.

### Circulating opsonins contribute to bacterial binding capacity

We next sought to characterize potential cell-extrinsic mechanisms for the enhanced bacterial binding capacity we identified in whole-blood cultures from children in the SAM versus healthy control group. Using plasma samples from the same cohort, we quantified circulating opsonins, soluble mediators that bind to microbes to enhance their detection and phagocytosis by immune cells. We found evidence for significantly higher plasma concentrations of innate opsonins for LPS in the SAM versus healthy control group (fig. S5; sCD14 coefficient: 0.34, 95% CI: 0.05 to 0.62, *P* = 0.020; LBP coefficient: 0.97, 95% CI: 0.67 to 1.26, *P* < 0.001). In contrast, we found weak evidence that plasma concentrations of the opsonizing antibody endotoxin LPS-specific immunoglobulin G (IgG; EndoCAb) were lower in the SAM group versus controls (fig. S5; coefficient: −0.31, 95% CI: −0.62 to 0.00, *P* = 0.052) and no evidence for a difference in total IgA titers between groups (fig. S5; coefficient: −0.25, 95% CI: −0.98 to 1.49, *P* = 0.688) in the subset of participants with available plasma for these assays (heathy controls, *n* = 36; SAM cases, *n* = 54).

Across nutritional groups, there were significant positive pairwise correlations between sCD14 and LBP (coefficient: 0.25, *P* < 0.001). Plasma sCD14 concentrations had a significant positive pairwise correlation with percentages of *E. coli*^+^ neutrophils (coefficient: 0.15, *P* = 0.034), lymphocytes (coefficient: 0.26, *P* < 0.001), and monocytes (coefficient: 0.20, *P* = 0.005; table S5). LBP also had a significant positive correlation with *E. coli*^+^ percentages of neutrophils (coefficient: 0.28, *P* < 0.001), lymphocytes (coefficient: 0.23, *P* = 0.001), and monocytes (coefficient: 0.24, *P* = 0.001) and was negatively correlated with meanFI for lymphocytes (coefficient: −0.19, *P* = 0.008; table S5). EndoCAb was not correlated with levels of other opsonins (sCD14, coefficient: −0.11, *P* = 0.301; LBP, coefficient: −0.06, *P* = 0.556; IgA, coefficient: −0.02, *P* = 0.821) but had a significant positive pairwise correlation with *E. coli* meanFI for lymphocytes (coefficient: 0.28, *P* = 0.006) but not for neutrophils (coefficient: −0.06, *P* = 0.542) or monocytes (coefficient: −0.15, *P* = 0.156). We did not find evidence to support a correlation between EndoCAb titers and percentages of *E. coli*^+^ lymphocytes, monocytes, or neutrophils, nor between total IgA and any bacterial binding readouts (table S5).

### SAM reduces monocyte activation and proinflammatory cytokine secretion in response to bacteria

Up-regulation of major histocompatibility complexes (e.g., HLA-DR) and co-receptors (e.g., CD86 and CD40) required for activation of T cells and secretion of soluble mediators to mobilize antibacterial functions in other cells and tissues, recruit immune cells, and promote bactericidal reactive oxygen species (ROS) production are key features of the innate immune response to bacterial infection. We quantified these characteristics in immune cells and supernatants from parallel 24-hour whole-blood cultures conducted without stimulus (negative controls) and with bacterial PAMP [*E. coli* LPS and heat-killed *S. typhimurium* (HKST)]. Unstimulated whole-blood cultures from children with SAM had higher percentages of neutrophils and a more mature monocyte phenotype (i.e., higher percentage of CD14^lo^ monocytes) than those from healthy controls (fig. S6, A and B, and table S3); culture duration shapes relative CD14/CD16 expression, and therefore, classical, intermediate, and nonclassical monocyte subsets were not evaluated in 24-hour whole-blood cultures. Unstimulated total monocytes from children with SAM expressed lower levels of HLA-DR (medianFI) than healthy controls but similar levels of CD86 and CD40 (fig. S6, C and E, and table S6). Basal concentrations of proinflammatory cytokines (IL-6, IL-8, and TNFα) in unstimulated whole-blood culture supernatants were similar in the SAM and healthy control groups (fig. S6F and table S6). Basal concentrations of MPO, an enzyme that catalyzes production of ROS, were higher in culture supernatants from the SAM versus healthy control group (fig. S6F and table S6).

**Fig. 2.**
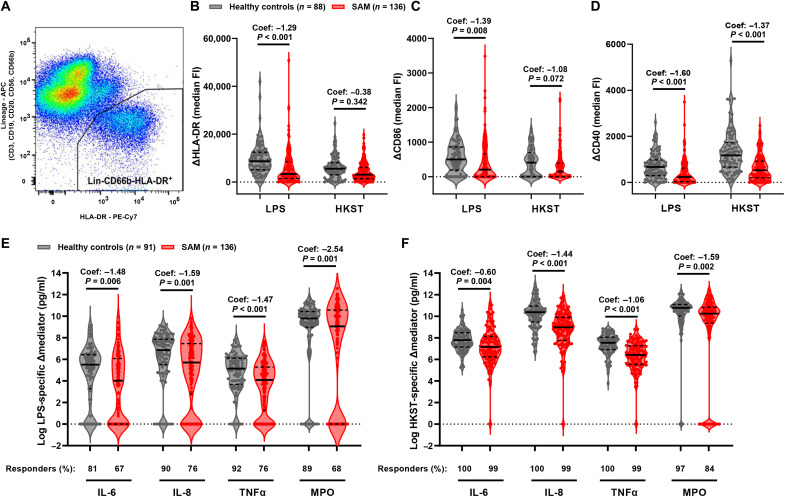
SAM compromises monocyte maturation and proinflammatory mediator secretion in response to bacterial PAMP. Whole-blood samples from children with SAM (red) and healthy controls (gray) were incubated for 24 hours in medium alone, LPS, or HKST; cultured cells were analyzed by flow cytometry (A to D; healthy controls, *n* = 88; SAM, *n* = 136), and culture supernatants were analyzed by ELISA (E and F; healthy controls, *n* = 91; SAM, *n* = 136). (**A**) Flow cytometry gating of total monocytes after exclusion of cells expressing markers of lymphocyte (Lin^+^) and granulocyte (CD66b^+^) lineage; representative example. PAMP-induced medianFI of (**B**) ΔHLA-DR-PECy7, (**C**) ΔCD86-FITC, and (**D**) ΔCD40-PerCP-Cy5.5 of total monocytes after subtraction of medianFI of monocytes in unstimulated cultures. (**E**) LPS-induced and (**F**) HKST-induced ΔIL-6, ΔIL-8, ΔTNFα, and ΔMPO after subtraction of concentrations in supernatants from unstimulated cultures; the percentage of children in each group who produced mediator concentrations greater than those present in unstimulated cultures (i.e., responders) are indicated. Violin plots indicate median (dark line) and IQR (dashed lines); unadjusted coefficients (Coef.) and *P* values are reported for univariable tobit regression by group.

To account for unstimulated differences in monocyte composition and basal activation marker expression between the two groups and focus on the capacity of immune cells to respond to a new bacterial challenge in vitro, we calculated PAMP-induced cell surface marker expression and mediator secretion for each child by subtracting their basal HLA-DR, CD86, and CD40 medianFIs and mediator concentrations present in unstimulated cultures from those in corresponding LPS- and HKST-stimulated whole-blood cultures; differences (Δ) in medianFI/mediator concentration were censored at zero (i.e., no increase in response to PAMP). Monocytes from children with SAM had lower LPS-induced ΔmedianFI for HLA-DR, CD86, and CD40 than those from healthy controls (Fig. 2, A to D; unadjusted coefficients and *P* values are indicated, and full details of analyses are in table S6). The same pattern was observed for HKST-induced monocyte surface marker ΔmedianFI but with stronger evidence for a difference in HKST-induced CD40 than for HKST-induced HLA-DR and CD86 (Fig. 2, B to D, and table S6). LPS- and HKST-induced ΔIL-6, ΔIL-8, ΔTNFα, and ΔMPO concentrations were all lower in supernatants from cultures conducted with blood samples from SAM versus healthy controls (Fig. 2, E and F, and table S6). There were a higher percentage of responders (i.e., produced PAMP-induced mediator concentrations greater than those present in unstimulated cultures) in the healthy control versus SAM group (Fig. 2, E and F), indicating that children with SAM are less able to respond to PAMP as well as producing lower concentrations of PAMP-induced mediators.

### Monocytes and neutrophils from children with SAM prioritize bacterial containment over systemic immune activation

**Fig. 3.**
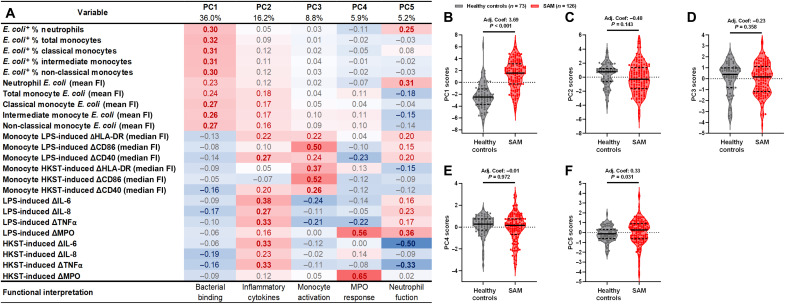
Monocytes and neutrophils of children with SAM prioritize bacterial containment over systemic immune activation. (**A**) Factor loadings for all antibacterial innate immune function variables included in a PCA (126 children with SAM and 73 healthy controls with full data on all variables). Table color-coded by strength and direction of association between the original variables and the resulting PC, with red indicating positive (bold for coefficient ≥ 0.3) and blue negative (bold for coefficient ≤ −3) association. Percentages correspond to the amount of variance in the dataset accounted for by each PC. Functional interpretation reflects the combination of the original variables captured by each PC. Scores for (**B**) PC1, (**C**) PC2, (**D**) PC3, (**E**) PC4, and (**F**) PC5 for each child in the SAM (red) and healthy control (gray) groups. Violin plots indicate median (dark line) and IQR (dashed lines); adjusted coefficients (Adj. Coef.) and *P* values are reported for multivariable linear regression by SAM status, adjusting for sex, age group, HIV infection status, hospital site, and immunoassay on the day of discharge.

Having identified differences in individual readouts of antibacterial innate immune cell function between the SAM and healthy control groups, we sought to characterize integrated patterns of neutrophil and monocyte function (i.e., *E. coli* meanFI and percentages of *E. coli*^+^ neutrophils, monocytes, and monocyte subsets from bacterial binding assays; LPS- and HKST-induced ΔHLA-DR, ΔCD86, and ΔCD40 medianFIs for total monocytes; and ΔIL-6, ΔIL-8, ΔTNFα, and ΔMPO in supernatants from whole-blood cultures) that explained the immune heterogeneity in our cohort via principal components analysis (PCA). One hundred twenty-six of 141 children (89.4%) in the SAM group and 73 of 92 children (79.3%) in the healthy control group had complete data for all variables, which were included in a single PCA. We identified five groups of antibacterial monocyte and neutrophil functions [principal components (PCs); Fig. 3A]: PC1 scores for each participant corresponded to their neutrophil and monocyte bacterial binding capacity; PC2 scores to their LPS- and HKST-induced proinflammatory cytokine (ΔTNFα, ΔIL-6, and ΔIL-8) release; PC3 scores to their LPS- and HKST-induced monocyte maturation marker expression (ΔHLA-DR, ΔCD86, and ΔCD40); PC4 scores to their LPS- and HKST-induced ΔMPO; and PC5 scores to a combination of their neutrophil bacterial binding and PAMP-induced ΔMPO release (Fig. 3A). PC1 to PC5 collectively captured 72.1% of the total variance in our immune function data.

We then tested our hypothesis that antibacterial innate immune cell function differs between children admitted to hospital with SAM relative to healthy controls from the same community using linear regression of participant scores for each PC. Unadjusted patterns of PC scores by SAM status were consistent with those reported for individual readouts of immune functions (table S7 and Figs. 1 and 2) with the advantage of integrating data from these inherently cross-regulatory and nonindependent variables and reducing false-positive error rates in statistical comparisons. We then adjusted analyses of PC scores by SAM status for characteristics that might affect both a child’s nutritional status and their immune function (i.e., age group, sex, HIV infection status, hospital site, and assessment of immune function on the same day as hospital discharge; Fig. 3, B to F, and table S7). Adjusted analyses provide evidence for a total effect of SAM status on innate immune cell bacterial binding capacity (PC1 scores; adjusted coefficient: 3.69, 95% CI: 2.97 to 4.40, *P* < 0.001) and neutrophil antibacterial response (PC5 scores; adjusted coefficient: 0.33, 95% CI: 0.03 to 0.63, *P* = 0.031), consistent with a shift in innate immune cell function favoring containment of bacteria by binding/phagocytosis. Despite having higher circulating and intestinal proinflammatory biomarker levels relative to healthy controls (Table 1 and fig. S5) and evidence that critically unwell children hospitalized in LMIC may have enhanced LPS-induced proinflammatory cytokine secretion (*39*), there was limited evidence that PC2 scores (adjusted coefficient: −0.48, 95% CI: −1.13 to 0.16, *P* = 0.143), PC3 scores (adjusted coefficient: −0.23, 95% CI: −0.73 to 0.27, *P* = 0.358), or PC4 scores (adjusted coefficient: −0.01, 95% CI: −0.39 to 0.38, *P* = 0.972) differed by nutritional status after adjusting for confounders (Fig. 3, C to E).

**Fig. 4.**
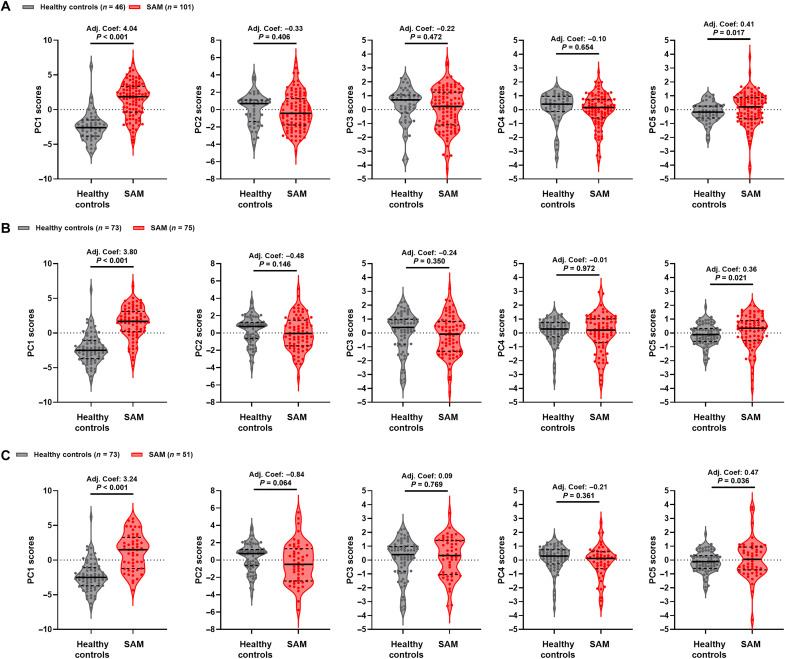
SAM differentiates innate antibacterial immune cell function from healthy controls among HIV-negative children and among children who had immune assessment at different times during hospitalization. Scores for PC1, PC2, PC3, PC4, and PC5 for (**A**) HIV-negative children in the healthy control (*n* = 46; gray) and SAM (*n* = 101; red) groups, (**B**) all healthy controls (*n* = 73) and SAM cases assessed before their day of discharge (*n* = 75), and (**C**) all healthy controls (*n* = 73) and SAM cases assessed on their day of discharge (*n* = 51). Violin plots indicate median (dark line) and IQR (dashed lines); adjusted coefficients (Adj. Coef.) and *P* values are reported for multivariable linear regression by SAM status, adjusting for variation due to sex, age group, hospital site, and immunoassay on the day of discharge (A) or sex, age group, hospital site, and HIV infection status (B and C).

Because HIV infection can influence many aspects of SAM and has a profound impact on the immune system, we undertook sensitivity analysis including only the HIV-negative children [Fig. 4A and table S8; SAM, *n* = 101 of 126 (80.2%); healthy controls, *n* = 46 of 73 (63.0%)]; this strengthened the association between SAM status and PC1 (adjusted coefficient: 4.04, 95% CI: 3.17 to 4.91, *P* < 0.001) and PC5 (adjusted coefficient: 0.41, 95% CI: 0.07 to 0.76, *P* = 0.017). Furthermore, because some children in the SAM group had their first immune function assessments on the day of hospital discharge when inpatient treatment had stabilized their acute clinical complications, we undertook two additional sensitivity analyses: (i) including only children who had their immune function assessed before the day of discharge [Fig. 4B and table S8; SAM, *n* = 75 of 126 (59.5%); healthy controls, *n* = 73 of 73 (100%)] and (ii) including only children assessed on the day of discharge [Fig. 4C and table S8; SAM, *n* = 51 of 126 (40.5%); healthy controls, *n* = 73 of 73 (100%)]. These analyses slightly weakened the effect size but did not affect the direction or significance of the association between SAM and PC1 and PC5 scores (Fig. 4 and table S8). There remained limited evidence to support an association between SAM status and PC2 to PC4 scores in sensitivity analyses (Fig. 4 and table S8).

We then tested whether the difference between SAM cases and healthy controls in PCs reflecting domains of innate immune cell bacterial binding capacity (PC1 and PC5) were influenced by plasma opsonins positively correlated with individual readouts of bacterial binding (i.e., sCD14, LBP, and EndoCAb); the association between both PCs and SAM status was retained in models adjusting for circulating CD14 (PC1 adjusted coefficient: 3.84, 95% CI: 3.10 to 4.57, *P* < 0.001; PC5 adjusted coefficient: 0.33, 95% CI: 0.00 to 0.66, *P* = 0.053), LBP (PC1 adjusted coefficient: 3.83, 95% CI: 3.00 to 4.66, *P* < 0.001; PC5 adjusted coefficient: 0.48, 95% CI: 0.13 to 0.82, *P* = 0.007), or EndoCAb (PC1 adjusted coefficient: 4.80, 95% CI: 3.93 to 5.67, *P* < 0.001; PC5 adjusted coefficient: 0.52, 95% CI: 0.12 to 0.92, *P* = 0.012) alongside age group, sex, HIV infection status, hospital site, and assessment of immune function on the same day as hospital discharge. Thus, despite their opsonizing role, differences in circulating opsonins were not sufficient to explain the higher PC1 and PC5 scores in the SAM versus healthy control group.

### Antibacterial innate immune cell function is partially shaped by age group, HIV infection, and wasting severity at hospital admission

Children with complicated SAM experience a range of comorbidities and risk factors for adverse clinical outcomes (*4*, *6*–*9*), which could plausibly affect their immune function relative to healthy controls. To explore this hypothesis, we used cross-fit partialing-out lasso linear regression to estimate the effect of variables associated with mortality in the HOPE-SAM cohort [i.e., HIV infection status, nutritional edema, age group, MUAC at baseline, and symptoms of infection at the time of immune function assessment (*6*, *9*)] on PC1 to PC5 scores of the SAM group. Other potential sources of immune heterogeneity in the SAM group were offered to the model as control variables [i.e., sex, hospital site, time to immunoassay (days), immune assessment on the day of discharge, HAZ, WHZ, cerebral palsy status, and birthweight (grams)]. In total, 109 of 126 children in the SAM group (86.5%) had complete data on all clinical and demographic variables for these analyses.

**Fig. 5.**
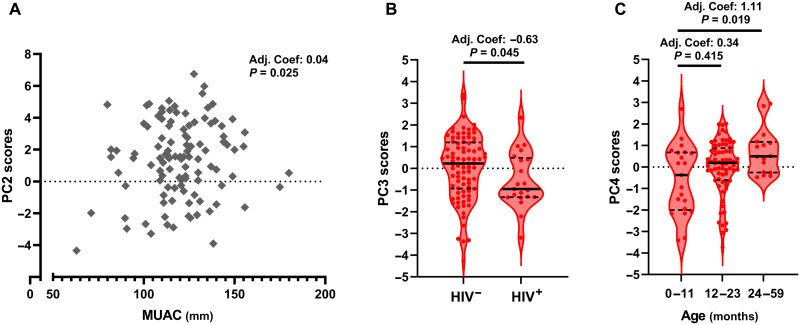
Clinical and demographic variables associated with adverse clinical outcomes from SAM shape some antibacterial innate immune cell functions. (**A**) PC2 (antibacterial inflammatory cytokines) scores plotted against MUAC (millimeters), (**B**) PC3 (antibacterial monocyte activation) scores plotted by HIV status, and (**C**) PC4 (antibacterial MPO) plotted by participant age group for the SAM group (*n* = 109). Adjusted coefficients (Adj. Coef.) and *P* values are reported for multivariable cross-partialing out lasso linear regression of PC scores by clinically relevant patient characteristics, adjusting for other clinically relevant variables (baseline edema and presence of any symptom of infection at the time of immune function assessment) and model-selected controls. Only exposure variables where there was evidence for an effect on immune function are shown; full analysis is provided in Table 2.

We did not find evidence to support an independent effect of clinically important variables on PC1 or PC5 scores (Table 2). However, we found evidence to support a positive association between PC2 scores and baseline MUAC (adjusted coefficient: 0.04, 95% CI: 0.00 to 0.07, *P* = 0.025; Fig. 5A), a negative association between PC3 scores and HIV infection (adjusted coefficient: −0.63, 95% CI: −1.25 to −0.01, *P* = 0.045; Fig. 5B), and a positive association between PC4 scores and age group; children in the 12- to 23-month (adjusted coefficient: 0.34, 95% CI: −0.47 to 1.15, *P* = 0.415) and 24- to 59-month (adjusted coefficient: 1.11, 95% CI: 0.18 to 2.05, *P* = 0.019) age groups had higher PC4 scores than the youngest age group (0 to 11 months; Fig. 5C). The best evidence that clinically important exposure variables influenced antibacterial innate immune cell function came from the PC4 model (Wald χ^2^: 13.72, *P* = 0.033) with weaker evidence from models for the other PCs (Table 2).

**Table 2.** Cross-fit partialing-out lasso regression of antibacterial innate immune function profiles by demographic and clinical variables associated with inpatient and postdischarge mortality among children with SAM.

		PC1*^,†^	PC2*^,‡^	PC3*^,§^	PC4*^,¶^	PC5*^,#^
Baseline age group						
0–11 months		Reference				
12–23 months	Adj. Coef.	0.21	−0.62	0.44	0.34	0.29
	95% CI	−1.03 to 1.46	−1.67 to 0.43	−0.42 to 1.29	−0.47 to 1.15	−0.30 to 0.88
	*P*	0.735	0.244	0.318	0.415	0.336
24–59 months	Adj. Coef	0.15	−0.83	0.48	1.11	0.20
	95% CI	−1.29 to 1.60	−2.10 to 0.44	−0.51 to 1.47	0.18 to 2.05	−0.57 to 0.98
	*P*	0.834	0.200	0.339	0.019	0.606
HIV status						
Negative		Reference				
Positive	Adj. Coef.	−0.81	0.49	−0.63	−0.18	0.50
	95% CI	−1.81 to 0.19	−0.40 to 1.39	−1.25 to −0.01	−0.95 to 0.59	−0.13 to 1.13
	*P*	0.114	0.281	0.045	0.650	0.122
Baseline edema status						
Non-edematous		Reference				
Edematous	Adj. Coef.	0.13	0.13	0.14	0.46	−0.10
	95% CI	−0.94 to 1.20	−0.85 to 1.12	−0.50 to 0.77	−0.18 to 1.10	−0.62 to 0.43
	*P*	0.809	0.789	0.671	0.163	0.719
Symptom/s of infection at time of immunoassay						
None		Reference				
≥1 symptom	Adj. Coef.	0.12	0.35	−0.08	−0.05	0.16
	95% CI	−0.74 to 0.98	−0.40 to 1.10	−0.61 to 0.46	−0.58 to 0.47	−0.28 to 0.61
	*P*	0.779	0.366	0.782	0.840	0.471
Baseline MUAC (mm)	Adj. Coef.	−0.02	0.04	0.00	0.01	0.00
	95% CI	−0.06 to 0.02	0.00 to 0.07	−0.02 to 0.02	−0.01 to 0.03	−0.03 to 0.04
	*P*	0.291	0.025	0.805	0.534	0.847

*n = 109 children with SAM; fixed exposure variables selected based on HOPE-SAM mortality and morbidity outcomes (*6*, *9*); controls offered to model: sex, hospital site, time to immunoassay, immune function assessment on the day of hospital discharge, baseline WHZ, baseline HAZ, birthweight, and cerebral palsy status

†Five controls, Wald χ^2^: 3.33, *P* = 0.767.

‡Three controls, Wald χ^2^: 7.90, *P* = 0.246.

§Three controls, Wald χ^2^: 7.00, *P* = 0.321.

¶Three controls, Wald χ^2^: 13.72, *P* = 0.033.

#Four controls, Wald χ^2^: 3.94, *P* = 0.685.

To explore whether the negative association between PC3 and HIV infection was also evident in HEU children, we reran this model for children with available data on exposure to maternal HIV infection (table S9). Consistent with the original model, children with HIV had significantly lower PC3 scores than HIV-unexposed uninfected children (HUU) (adjusted coefficient: −0.65, 95% CI: −1.28 to −0.03, *P* = 0.041). HEU children had lower PC3 scores than HUU, but this difference was not statistically significant (adjusted coefficient: −0.10, 95% CI: −0.93 to 0.73, *P* = 0.817).

### Antibacterial innate immune cell function converges to that of healthy children with duration of inpatient rehabilitation

**Fig. 6.**
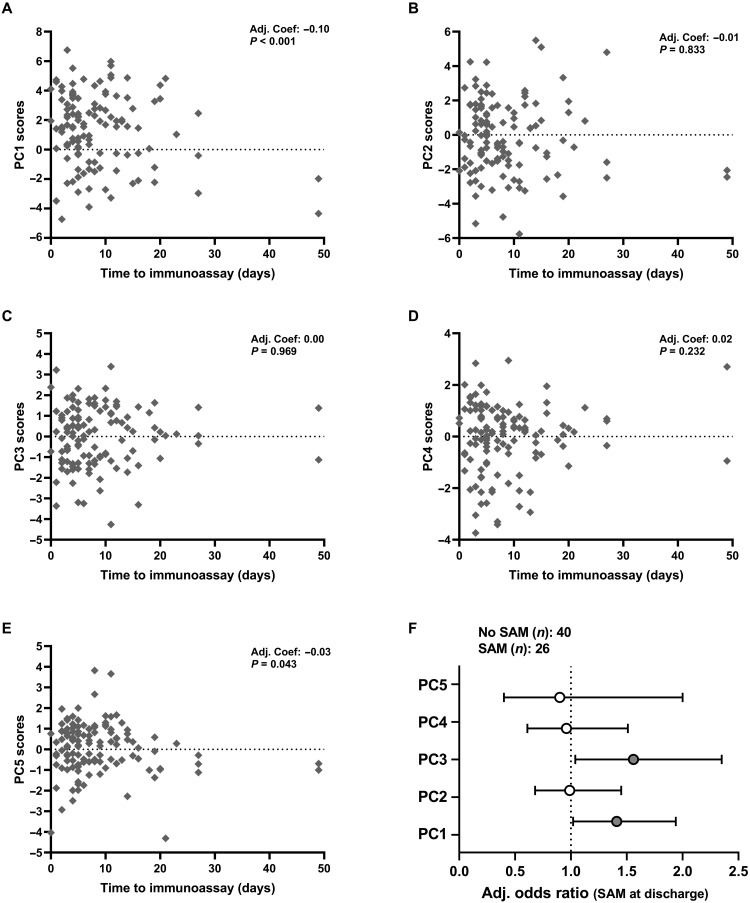
Antibacterial innate immune cell function of children with SAM converges toward that of healthy controls during inpatient rehabilitation and is associated with persistent SAM at hospital discharge. Cross-sectional scores for (**A**) PC1, (**B**) PC2, (**C**) PC3, (**D**) PC4, and (**E**) PC5 for children with SAM (*n* = 120) plotted against the time between their initial clinical assessment at hospital admission and immune function assessment (days); adjusted coefficients (Adj. Coef.) and *P* values are reported for multivariable linear regression by time to immunoassay, adjusted for hospital site, HIV infection status, edema status at baseline, symptom/s of infection on the day of blood sampling, and immunoassay on the day of discharge. (**F**) Odds ratios for persistent SAM at discharge among children who had immune function assessment before the day of discharge (*n* = 66); logistic regression, adjusted for variation due to sex, age group at baseline, HIV infection status, hospital site, edema at baseline, and baseline MUAC.

Inpatient rehabilitation of children with complicated SAM aims to resolve clinical complications, including acute infections, and begin nutritional stabilization so that children can be discharged to outpatient community management of SAM (*2*). Given that all children in the SAM group survived to discharge, we hypothesized that those who had a longer period of inpatient rehabilitation before their immune function assessment (time to immunoassay) would have innate immune cell function more similar to healthy controls than those assessed at hospital admission. We found cross-sectional evidence for a negative association between PC1 (Fig. 6A; adjusted coefficient: −0.10, 95% CI: −0.15 to −0.05, *P* < 0.001) and, to a lesser extent, PC5 (Fig. 6E; adjusted coefficient: −0.03, 95% CI: −0.05 to 0.00, *P* = 0.043) and time to immunoassay after adjusting for hospital site, HIV infection status, baseline edema status, symptoms of acute infection on the day of immunoassay, and immune function assessment on the day of discharge (table S10). We did not find substantial evidence that antibacterial proinflammatory cytokine secretion (PC2; adjusted coefficient: −0.01, 95% CI: −0.06 to 0.01, *P* = 0.833), up-regulation of monocyte activation markers in response to bacterial PAMP (PC3; adjusted coefficient: 0.00, 95% CI: −0.04 to 0.04, *P* = 0.969), or PAMP-induced MPO production (PC4; adjusted coefficient: 0.02, 95% CI: −0.02 to 0.06, *P* = 0.232) changed with time to immunoassay (Fig. 6, B to D, and table S10).

### Antibacterial immune function during hospitalization is associated with persistent SAM at discharge

In the HOPE-SAM cohort, children with persistent SAM at discharge were at greater risk of subsequent mortality and had persistently lower WHZ and MUAC throughout 1 year of postdischarge follow-up than children who were discharged with moderate acute malnutrition (MAM) or adequate nutrition (*9*). In light of our observations that children with SAM had a distinct bacterial binding capacity to healthy controls, and cross-sectional evidence that immune function of children in the SAM group converges toward that of the healthy group during inpatient rehabilitation, we hypothesized that immune function would be associated with persistent SAM at hospital discharge. We tested this hypothesis in children with SAM who had immune function assessment before the day of discharge and who were not discharged against medical advice (*n* = 66); 26 of 66 (39.4%) had persistent SAM and 40 of 66 (60.6%) had MAM (−2 < WHZ < −1; *n* = 13) or adequate nutritional status (WHZ > −1; *n* = 27) at discharge (table S11).

Unadjusted logistic regression provided limited evidence for antibacterial innate immune function (PC1 to PC5) affecting odds of persistent SAM at discharge (fig. S7 and table S12). However, after adjusting for confounders (sex, hospital site, HIV infection status, baseline age group, baseline edema status, and baseline MUAC), higher scores for both PC1 (bacterial binding capacity) and PC3 (monocyte activation) were associated with 1.41 (95% CI: 1.02 to 1.94; *P* = 0.039) and 1.56 (95% CI: 1.04 to 2.35; *P* = 0.032) higher odds of persistent SAM at discharge, respectively (Fig. 6F).

## DISCUSSION

Complicated SAM has typically been considered an immunosuppressed state, with functional responses to bacteria thought to reflect the immunoparalysis phenotype described in sepsis and multiple organ dysfunction syndrome (*32*, *40*, *41*). Previous pilot studies have found conflicting evidence for the capacity of innate immune cells to bind/phagocytose, up-regulate activation markers, secrete immune mediators, initiate oxidative burst, and kill bacteria in undernourished children (*34*, *35*). By characterizing multiple domains of innate immune cell function simultaneously to reflect their inherent interactions in a cohort powered for comparison to community-relevant healthy controls, our data demonstrate that children with SAM who survive to hospital discharge achieve a compromise rather than a global deficiency in antibacterial innate immune cell function. We show that innate immune cells from children with SAM share some of the hallmark features of immunoparalysis, namely, lower expression of HLA-DR and costimulatory molecules (CD86 and CD40) by monocytes, and lower proinflammatory cytokine production upon in vitro PAMP challenge (PCs 2 to 4). This accords with a previous study among 37 children admitted to the University Teaching Hospital, Lusaka with complicated SAM, which found that blood dendritic cells had impaired IL-12 responses to LPS at hospital admission and a small percentage of children (17%; *n* = 6) failed to up-regulate HLA-DR upon LPS stimulation (*42*). However, in parallel with lower monocyte activation/maturation and proinflammatory mediator secretion, we show that monocyte and neutrophil bacterial binding capacity (PC1 and PC5) was markedly higher in children with SAM compared to healthy controls. Given the association between elevated systemic proinflammatory mediators and mortality from complicated SAM (*15*, *26*, *27*), it is plausible that favoring bacterial containment via enhanced binding/phagocytosis over cytokine secretion by innate immune cells would provide a survival advantage during acute hospital admission. A similar adaptive balance has been observed for monocytes derived from adults hospitalized with Gram-negative sepsis (*n* = 3), which had lower expression of monocyte activation, proinflammatory cytokine and chemokine genes coinciding with enhanced phagocytosis, and growth inhibition of live *E. coli* in vitro before versus after recovery (*28*). While sepsis provides a pertinent analog for the systemic endotoxin exposure observed in SAM and a sepsis-like biomarker profile has been described among children recovering from SAM who die after hospital discharge (*26*, *27*), only one (0.01%) of our cohort of children with complicated SAM was septic at the time of blood sampling. Thus, improved understanding of how immune adaptations occur could support stratification of patients according to specific immunological risk factors, as has been trialed for immune hyper- versus hyporeactive states in septic patients (*43*, *44*).

Recent evidence from experimental animal models supports our data that pathogen binding by innate immune cells is enhanced in the context of undernutrition; cutaneous monocytes from mice fed polynutrient-deficient diets more readily internalized *Leishmania donovani* parasites and more *L. donovani*^+^ monocytes trafficked to the spleen relative to nutrient-sufficient animals (*45*). In this study, we used a whole blood–based in vitro assay of bacterial binding capacity, including plasma components alongside all circulating cell types; the enhanced bacterial binding capacity that we observe in SAM could therefore reflect cell-intrinsic differences in activation, trafficking, and expression of phagocytic receptors and/or increased levels of cell-extrinsic circulating bacterial binding proteins that accelerate bacterial uptake. Our data implicate innate opsonins (sCD14 and LBP) in the greater proportions of innate immune cells capable of binding to *E. coli* bioparticles and their greater binding intensity in the context of SAM, and adaptive opsonins (EndoCAb) in the increased intensity of lymphocytes but not innate immune cell binding. Although not phagocytic receptors per se, LBP and sCD14 bind Gram-negative bacteria, act as co-receptors for LPS-TLR4 (Toll-like receptor 4) interactions on the cell surface, mediate phagocytosis in some settings, and confer responsiveness to bacteria by binding to the membranes of nonmonocytic (CD14^−^) cells in complex with LPS (*46*–*48*). The SAM group also had higher percentages of intermediate monocytes, which mature from classical monocytes and have higher expression of the Fcγ III receptor (CD16), which enhances phagocytosis and degranulation in response to IgG-opsonized bacteria (*49*), indirect evidence that SAM promotes expansion of monocyte subsets with higher putative avidity for IgG. Adjusting for innate and adaptive opsonins did not abrogate differences in PC1 or PC5 between the SAM and healthy control groups, supporting a role for immune cell–intrinsic factors as well as circulating opsonins in the enhanced bacterial binding capacity of children with SAM. These mechanisms and how they relate to subsequent immune cell trafficking, generation of adaptive immunity, and infectious susceptibility warrant further study.

It is unclear whether previously described differences in immune phenotype in children admitted with complicated SAM reflect nutritional status and/or specific patient characteristics/comorbidities (*34*). In the HOPE-SAM cohort, mortality over 1 year of postdischarge follow-up was associated with HIV infection [adjusted hazard ratio (HR): 3.83; 95% CI: 2.15 to 6.82], persistent SAM at discharge (adjusted HR: 2.28; 95% CI: 1.22 to 4.25), cerebral palsy (adjusted HR: 5.60; 95% CI: 2.72 to 11.50), and non-edematous SAM at admission (adjusted HR: 2.23; 95% CI: 1.24 to 4.01) (*9*). Young age, SAM at discharge, non-edematous SAM at admission, and cerebral palsy were also associated with hospital readmission or poor nutritional recovery after discharge (*6*). Symptoms of acute infection have previously been implicated in inpatient mortality among children hospitalized with SAM (*4*). We did not find evidence that these risk factors for poor clinical outcomes explained variation in scores for PC1 or PC5, the domains of immune function that best differentiated SAM cases from healthy controls. Thus, enhanced bacterial binding by innate immune cells from children with SAM is not solely due to their complications/comorbidities and may reflect as-yet-uncharacterized immune activating/regulatory processes specific to SAM. However, our data also demonstrate that demographic and clinical heterogeneity in children with SAM can be more influential for other domains of antibacterial innate immune cell function. PC2 was positively associated with MUAC, which reflects findings from a recent study among children admitted to hospitals in Kenya and Uganda with all-cause critical illness (46.0% were severely wasted). In that study, LPS-induced whole-blood proinflammatory cytokine responses, including IL-6, IL-8, and TNFα, were lower among hospitalized versus healthy children in unadjusted analyses; this relationship was reversed after adjustment for MUAC (*39*), indicative of a distinct pattern of LPS-induced responses among wasted children.

HIV profoundly affects monocyte composition, trafficking, and function, including maturation of monocytes into intermediate and nonclassical subsets (*50*), circulating numbers due to increased homing to the gut (*51*), and release of sCD14 into circulation (*52*). HIV is also associated with the non-edematous form of SAM, a more severe pattern of wasting, and increased risk of opportunistic infections, and HIV-driven inflammation is a risk factor for hospitalization with SAM among children living with HIV in LMIC (*36*, *53*, *54*). Consistent with HIV having an impact on innate immune cell function, we found that excluding children with HIV strengthened the effect of SAM status on PC1 and PC5 in sensitivity analyses. HIV infection and, to a lesser extent, HIV exposure were also associated with lower monocyte activation in response to bacterial PAMP (PC3) within the SAM group. PC4 scores were lowest in the youngest age group, suggesting that these children were less able to release MPO, an enzyme that catalyzes oxidative burst that is most abundantly expressed in neutrophil granules, upon bacterial PAMP stimulation. All children with SAM had higher basal MPO levels in unstimulated culture supernatants compared to healthy controls, but basal levels were highest among children with SAM in the youngest age group (fig. S8), indicating that they had higher systemic oxidative stress and neutrophil degranulation relative to children in the older two age groups. Preexposure of monocytes from healthy adults to LPS in vitro (*n* = 2) impairs their cytokine release, oxidative burst, and pathogen killing compared to saline-treated monocytes (*n* = 3) (*55*), a potential mechanism for how heightened oxidative stress in the youngest age group might compromise their defense against subsequent infections.

We did not find evidence that edema or coincident symptoms of infection influenced any of the immune function PCs. These were unexpected findings given that edema is positively associated with circulating levels of the extracellular matrix (ECM) protein lumican, and ECM remodeling mediators matrix metalloprotease 2 (MMP2), tissue inhibitors of MMP 1 (TIMP1), and TIMP2 (*56*), which might be expected to influence immune cell trafficking and function. Furthermore, acute infections expand and prime immune cells in vivo and 60.0% of our cohort had at least one symptom of infection. However, because we were reliant on physician examination–based identification of infections per standard of care at the hospital recruitment sites, our study may have underestimated the prevalence of infections. It is also plausible that reduced cytokine production and monocyte activation marker expression in response to bacterial PAMP might mask infectious symptoms in children with SAM. Inclusion of molecular diagnostics in future studies would provide greater scope to characterize the impact of concurrent infections and specific pathogens on SAM immune function.

Nutritional rehabilitation after hospital admission relies on resolution of clinical complications alongside therapeutic refeeding; an outstanding question that we are pursuing using longitudinal samples from this cohort during postdischarge follow-up is whether or not rehabilitation of immune cell function accords with anthropometric recovery (*36*). Cross-sectional evaluation during inpatient rehabilitation demonstrated that bacterial binding (PC1) and neutrophil responses (PC5) were negatively associated with the duration between hospital admission and immune function assessment. High scores for PC1 and PC5 differentiated children with SAM from healthy controls; therefore, their negative relationship with duration of hospitalization in the SAM group is indicative of these responses converging toward those seen in healthy immune cells as children recover. This has previously been suggested in pilot studies of blood immune cell function (*n* = 10) (*57*) and for circulating dendritic cells from children hospitalized with SAM in Zambia, which were higher in number, LPS-induced HLA-DR expression, and unstimulated IL-12 secretion at discharge than at hospital admission (*n* = 27) (*42*). Convergence of innate immune cell function in the SAM group toward that seen in healthy children could reflect and contribute to resolution of other physiological features disrupted in complicated SAM and posited as drivers of systemic inflammation (e.g., dysbiosis, metabolic profiles, and gut barrier function) (*25*, *27*, *58*–*60*). However, “immune rehabilitation” of antibacterial responses was incomplete because all but 9 of 126 (7.1%) of the SAM group had PC1 scores greater than the median score for the healthy group. Functional indicators of monocyte activation and mediator secretion in response to PAMP (PC2 to PC4) were unaffected by time to immunoassay, consistent with their stronger association with more stable participant characteristics (age group and HIV infection status) and continuous relationship with MUAC rather than with SAM status.

The clinical importance of antibacterial innate immune cell function to rehabilitation from SAM is supported by evidence that PC1 scores during hospitalization were associated with SAM status at discharge, a predictor of both mortality and persistent wasting over a year of postdischarge follow-up in HOPE-SAM (*6*, *9*). Scores for PC3 (PAMP-induced monocyte activation), which tended to be lower in children with SAM than healthy controls, were associated with higher odds of persistent wasting within the SAM group. Given the energetic cost of monocyte activation and turnover (*61*), which is associated with changes in circulating lipid levels (*61*), it is plausible that heightened proinflammatory capacity of blood monocytes might benefit antibacterial defense but compromise weight gain in children recovering from complicated SAM. The association between immune cell function and wasting that we identified accords with evidence from HIV-negative children recovering from SAM in Kenya for whom MUAC and WHZ gains over the first 60 days after discharge were negatively associated with circulating biomarkers of systemic inflammation (*59*).

While this study advances our understanding of immune function in SAM, our data should be interpreted in the context of several limitations. First, our cohort included only children able to provide a ≥2-ml blood sample, necessarily excluding the most acutely unwell children and those who died before enrolment or blood sampling; our findings are therefore most applicable to children who survive to hospital discharge. Second, we undertook immune function assessment across a range of time points after hospital admission, which contributed to immune heterogeneity in the SAM group. Adjusting analyses for time to immunoassay, presenting cross-sectional analyses of PCs by time to immunoassay and sensitivity analyses focusing on children for whom immune function was assessed before the day of discharge, go some way to demonstrate the effect of this variable on our findings and the generalizability of the SAM-related differences that we see despite this limitation. Third, there remains much to learn about the immunobiology of SAM (*24*, *35*), much of which was not possible to explore in this study but would be pertinent to our interpretation. We chose to focus on Gram-negative bacterial PAMP, which are among the most common clinical isolates from children with SAM but unlikely to be the only group of infections nor LPS the only PAMP translocated in this context; in our cohort alone, 11.5% of the SAM group had fungal infections (oral candidiasis) and 13.8% had suspected tuberculosis at the time of their immunoassay, and Gram-positive bacteria, viruses, fungi, and parasites are also prevalent in undernourished children (*10*, *15*). Fourth, although our study is the largest of its kind, powered for our primary comparison between the SAM and healthy groups, it is increasingly apparent from clinical studies, including our own (*6*, *9*, *53*), that specific comorbidities contribute to subconditions within current SAM diagnostic criteria (e.g., HIV-SAM, cerebral palsy–SAM, and edematous/non-edematous SAM); a larger or comorbidity-focused sample size would be required to conclusively evaluate the impact of these variables on immune function. Because some of these comorbidities are shared by hospitalized children without SAM and recent studies suggest that these children have distinct antibacterial proinflammatory mediator responses relative to healthy controls to those we observe in SAM (*39*, *62*), it would be desirable to include such children as an additional comparison group in future studies.

Overall, we provide evidence for a functional shift in how innate immune cells respond to bacterial PAMP during hospitalization with complicated SAM, with enhanced bacterial binding capacity of monocytes and neutrophils differentiating children with SAM from healthy controls alongside characteristics of impaired monocyte maturation and proinflammatory cytokine secretion to bacterial PAMP. Differences in bacterial binding capacity are not explained by patient demography or clinical comorbidities, but are more tractable to inpatient treatment than other domains of innate immune function and are associated with persistent SAM at discharge, a predictor of mortality and poor nutritional recovery after hospital discharge (*6*, *9*). Thus, innate immune cells from children with SAM strike a balance in their response to new infectious challenges, which affects inpatient rehabilitation and may contribute to persistent health deficits after hospital discharge.

## MATERIALS AND METHODS

### Study design

Immune function of children with SAM (IMMUNO-SAM) is a translational immunology study designed to characterize antibacterial innate immune cell function using blood samples from children under 5 years old hospitalized with complicated SAM at three tertiary referral hospitals in Lusaka (University Teaching Hospital), Zambia and Harare (Harare Central Hospital and Parirenyatwa Hospital), Zimbabwe. All participants were enrolled into a longitudinal observational study for which the primary outcome was mortality over 1 year of postdischarge follow-up (HOPE-SAM); clinical study protocols and outcomes are published elsewhere (*6*, *9*, *36*, *37*).

Ethical approval was obtained from the University of Zambia Biomedical Research Ethics Committee and the Medical Research Council of Zimbabwe. The ethics committee of the Queen Mary University of London provided an advisory review. Caregivers of all participants provided written informed consent for their participation.

### Study participants

SAM cases were children aged 0 to 59 months, admitted to one of the three hospital sites with complicated SAM. SAM was diagnosed according to the current WHO definition as WHZ < −3, MUAC < 115 mm and/or the presence of nutritional edema for children above 6 months of age, or WHZ < −3 and/or nutritional edema for those aged under 6 months (*2*). Healthy controls were children aged 6 to 59 months who were adequately nourished (WHZ > –1) and clinically well (no symptoms of acute illness). So that the control group included exposures relevant to an LMIC context, clinically well children with HIV infection were included in the healthy control group alongside those with conditions that are highly prevalent among children growing up in Zambia and Zimbabwe (e.g., HEU and stunting); all children with HIV in the healthy control group were on ART at the time of sampling.

Additional inclusion criteria for both groups were (i) enrolled into HOPE-SAM after ethical approval of IMMUNO-SAM procedures (August 2017), (ii) known HIV infection status (i.e., with or without infection), (iii) ≥2-ml blood sample collected during hospitalization (SAM group) or at baseline visit (healthy controls), and (iv) no underlying chronic gastrointestinal disease or a known malignancy. For SAM cases, any blood sample collected between their initial clinical assessment at baseline and their discharge from hospital was admissible to the study. Age groups were prespecified for the HOPE-SAM study (0 to 5 months, 6 to 11 months, 12 to 23 months, and 24 to 59 months) (*36*); the seven children in the SAM group who were aged under 6 months at admission were included in the youngest age group (0 to 11 months).

### Sample size

There was insufficient data with which to estimate a sample size for comparing immune function between SAM cases and controls when recruitment started; we therefore included all eligible children from study approval to the end of the HOPE-SAM recruitment window (March 2018) to maximize statistical power. We report 95% CIs for all analyses as a recognized alternative to post hoc power calculations (*63*), indicating that our study is adequately powered to detect between-group differences (i.e., small likelihood of type II error).

### Clinical procedures

Clinical, household, and demographic characteristics were collected from the primary caregiver of each child via questionnaire at study baseline, i.e., as soon as possible after hospital admission. Clinical care for SAM cases was implemented according to country-specific guidelines based on WHO guidelines (*2*, *3*). HIV infection statuses for children aged >18 months and their mothers, if present, were determined using a rapid antibody test algorithm, and for children <18 months using HIV DNA polymerase chain reaction. Where HIV test kits were not available at the study hospitals for testing by the clinical team, HIV testing was done by the HOPE-SAM study team. Daily clinical review of each child with SAM was undertaken by a study physician using a standardized inpatient review form. Anthropometry was conducted at baseline and repeated for SAM cases on the day of discharge. Weight was recorded to the nearest 10 g using a Seca384 electronic scale for infants and to the nearest 100 g using a Seca874 electronic scale for older children. MUAC was recorded to the nearest 1 mm using WHO/United Nations Children's Fund (UNICEF) MUAC tape. Length and height were measured to the nearest 0.1 cm using UNICEF measuring boards.

Children were discharged to outpatient community-based management of nutritional rehabilitation according to WHO-adapted country guidelines, which specify that discharge is appropriate when a child’s medical complications, including edema, are resolving, they are clinically well and alert, and their appetite has returned (*1*, *2*).

### Blood sample collection

Blood samples (1 ml/kg up to a maximum of 5.4 ml) were collected into endotoxin-free EDTA-treated blood collection tubes. Blood was not collected from children with severe anemia (known hemoglobin of <60 g/liter).

### Bacterial binding assay

On the day of blood sample collection, 100 μl of undiluted whole blood was added to preprepared cryovials with culture medium only [RPMI 1640 medium supplemented with 1% (v/v) penicillin-streptomycin; unstimulated control] and 5 × 10^5^ AF488-conjugated *E. coli* bioparticles diluted in culture medium; assay conditions were prepared, aliquoted, and cryopreserved in bulk to standardize stimulation conditions for all participants. Cultures were mixed thoroughly and incubated for 1 hour at 37°C, 6% CO_2_ using a CO2Gen compact system. After 1 hour, cultured cells were treated with 1× fluorescence-activated cell sorting (FACS) lysing and fixative buffer for 10 min, washed in phosphate-buffered saline (PBS), resuspended in cell freezing medium [PBS with 10% (v/v) dimethyl sulfoxide, 40% (v/v) heat-inactivated fetal bovine serum], and gradually frozen overnight at 1°C/min to −80°C using a Mr. Frosty freezing container. This assay has been validated in previous studies (*64*) and adapted from the manufacturer’s protocols (Life Technologies) to provide a simple combined measure of bacterial binding and phagocytosis. Commercially available pH-sensitive fluorochrome conjugates, which can distinguish between binding and phagocytosis, are quenched by cell fixation and were therefore unsuitable for our study where cells were fixed and analyzed in bulk to minimize technical variation over the 2-year study period. Details of key reagents are provided in table S13.

### Whole-blood cultures

On the day of blood sample collection, 750 μl of whole blood was diluted in 1.45 ml of culture medium. Diluted blood (500 μl per well) was added to preprepared 48-well culture plates containing culture medium alone (unstimulated control), HKST (final concentration: 1 × 10^8^ cells/ml), and ultrapure *E. coli* LPS (final concentration: 0.25 EU/ml). Whole-blood cultures were incubated for 24 hours at 37°C, 6% CO_2_ using the CO2Gen compact system. Cell-free culture supernatants were harvested and cryopreserved at −80°C. Cultured blood cells were harvested by gentle pipetting across the culture well surface to liberate adherent cells. Cultured cells were then subjected to red blood cell lysis, fixed, and cryopreserved, as above. Details of key reagents are provided in table S13.

### Plasma, stool, and culture supernatant ELISA

CRP, sCD14, sCD163, and LBP were quantified in plasma [Biotechne enzyme-linked immunosorbent assay (ELISA) kits, Slough, UK]; and MPO (Immundianostik, Bensheim, Germany) and neopterin (GenWay Biotech Inc., San Diego, CA, USA) were quantified in stool via ELISA at Zvitambo and TROPGAN laboratories; methods were previously reported (*6*, *9*, *36*). Titers of EndoCAb and total IgA were quantified in plasma via ELISA (reagent details are provided in table S13) at Queen Mary University of London.

TNFα, IL-6, IL-8, and MPO concentrations were quantified in whole-blood culture supernatants by ELISA (reagent details are provided in table S13) at Zvitambo and TROPGAN laboratories. ELISA lower limits of detection were as follows: TNFα, 15.6 pg/ml; IL-6, 4 pg/ml; IL-8, 31.2 pg/ml; and MPO, 62.5 pg/ml. Concentrations below the assay detection limit were censored at the limit of detection; those greater than the top standard concentration were rerun at higher dilution and multiplied by the dilution factor.

### Flow cytometry

Cryopreserved bacterial binding assay cell samples were labeled with the following fluorochrome-labeled antibody panel: Lin (CD3, CD19, CD20, CD56)–APC, CD66b-PerCPCy5.5, CD16-APCCy7, HLA-DR–PECy7, and CD14-PE (table S13) to identify which cell types were bound to the AF488-labeled *E. coli*–coated bioparticles. Cryopreserved whole-blood culture cell samples were labeled with the following fluorochrome-labeled antibody panel: Lin (CD3, CD19, CD20, CD56)–APC, CD66b-APC, CD16-APCCy7, HLA-DR–PECy7, CD14-PE, CD86–fluorescein isothiocyanate (FITC), and CD40-PerCPCy5.5 (table S13). Culture duration shapes relative CD14/CD16 expression, and therefore, classical, intermediate, and nonclassical monocyte subsets were quantified in unstimulated cells from bacterial binding assays (1 hour) but not whole-blood cultures (24 hours); CD14^hi^ (less mature) and CD14^lo^ (more mature) were used for monocyte phenotyping in unstimulated 24-hour cultures (Fig. 1). Flow cytometry was conducted on the day of antibody labeling using six-channel BD Biosciences FACSVerse cytometers (488- and 633-nm lasers) at Zvitambo and TROPGAN laboratories after daily instrument calibration using CS&T beads and the Performance QC program in FACSuite software. Cell samples were labeled and analyzed in batches, including samples from both SAM and healthy control groups in each batch. Flow cytometry gating strategies (fig. S2) were implemented in FlowJo version 10.8.1 (FlowJo LLC, USA); universal gates were based on fluorescence-minus-one controls for each marker, applied to all samples, and adjusted per participant based on their corresponding assay-specific unstimulated control sample. FlowJo analyses were conducted blind to nutritional status. Upon unblinding, two cell samples from whole-blood culture, which had insufficient cell numbers for flow cytometry analysis, were identified as belonging to participants in the healthy control group; the healthy group therefore has a lower sample size for cells (*n* = 88) than for supernatants (*n* = 91) from whole-blood cultures.

### Statistical analyses

Unadjusted analyses of individual assay readouts (i.e., fractional regression for proportional data; linear regression for log-transformed plasma biomarker concentrations and *E. coli* meanFI; tobit regression for log-transformed ΔHLA-DR, ΔCD86, and ΔCD40 medianFI; and log-transformed ΔIL-6, ΔIL-8, ΔTNFα, and ΔMPO) by group were presented for context as these readouts have not been previously reported for SAM.

tSNE plots of all compensated cell surface markers (Lin, CD14, CD16, CD66b, HLA-DR) in 1-hour bacterial binding assays were generated for 500 *E. coli*^+^ leukocyte events per participant for all SAM cases and healthy controls using the cytofkit package in R according to publically available pipelines and code (*65*). Data were entered directly from compensated. fcs files gated on total leukocytes, singlets, and *E. coli*^+^ per fig. S2A, and FlowSOM analysis was applied using the cytofkit graphical user interface with the following settings: data merging by ceil, data transformation by autoLgcl, dimensionality reduction by tSNE, data clustering by Rpehnograph, and perplexity 300.

The study primary analyses were linear regression of integrated variables reflecting patterns of innate immune cell function (PC1 to PC5) by SAM status, adjusted for clinical and demographic confounders. Log-transformed PAMP-induced innate immune cell function outcome measures for all SAM cases (*n* = 126) and healthy controls (*n* = 73) with complete data were standardized (mean = 0, SD = 1) and entered into a single PCA. Resulting PCs were selected for subsequent analysis of immune function profiles if they had an eigenvalue of >1 and had a factor loading of ≥0.3 or ≤−0.3 for at least one of the original innate immune function variables. Linear regression was used to analyze the effect of SAM status on PC1 to PC5 scores. Variables were selected for inclusion in adjusted linear regression model based on causal inference methodology using a directed acyclic graph of exposure and outcome measures generated using Daggity software (fig. S9A) (*66*). Variables included in the WHO case definition of the SAM [i.e., WHZ, MUAC, edema, and acute clinical complications (*1*)] were not adjusted for in these analyses because they co-vary with SAM status. We undertook sensitivity analyses by SAM status including only (i) HIV-negative participants, (ii) participants for whom immune function was assessed before the day of discharge, and (iii) participants for whom immune function was assessed on the same day as discharge.

The effects of clinically relevant demographic and clinical characteristics on antibacterial innate immune function within the SAM group were estimated for each PC using cross-fit partialing-out lasso linear regression with default lambda selection. Sex, hospital site, time to immunoassay, baseline WHZ, baseline HAZ, birthweight, and cerebral palsy status were offered as control variables based on their putative contribution to variation in both immune function and clinical outcomes (fig. S9B).

To characterize the cross-sectional relationship between immune function and duration of hospitalization within the SAM group, we analyzed PC1 to PC5 scores by time to immunoassay via linear regression, adjusting for hospital site, HIV infection status, baseline edema status, symptoms of infection on the day of immune function assessment, and immune function assessment on the day of discharge (fig. S9C).

We tested the effect of immune function assessed before the day of discharge on SAM status at discharge via logistic regression of PC scores within the SAM group, adjusting for sex, baseline age group, HIV infection status, baseline edema status, hospital site, time to immunoassay, and baseline MUAC (fig. S9D). Children discharged against medical advice were excluded from these analyses.

All statistical analyses were performed using STATA version 17 (StataCorp LLC, USA). Outcome variables were plotted using Prism version 9 (GraphPad Software Inc., USA) and R version 4.2.0.
