# Supplementary material for: Severe acute malnutrition promotes bacterial binding over proinflammatory cytokine secretion by circulating innate immune cells
Source: Sci Adv. 2023 Nov 1;9(44):eadh2284. doi: 10.1126/sciadv.adh2284 (PMC10619937; doi:10.1126/sciadv.adh2284)
Supplement: Supplementary file 1 — Figs. S1 to S9 Tables S1 to S13 [file sciadv.adh2284_sm.pdf]

Science Advances
AAAS

Supplementary Materials for

Severe acute malnutrition promotes bacterial binding over pro-inflammatory cytokine secretion by circulating innate immune cells

Tracy N. Phiri *et al.*

Corresponding author: Claire D. Bourke, c.bourke@qmul.ac.uk

Sci. Adv. **9**, eadh2284 (2023)
DOI: 10.1126/sciadv.adh2284

This PDF file includes:

Figs. S1 to S9
Tables S1 to S13

SUPPLEMENTARY FIGURES

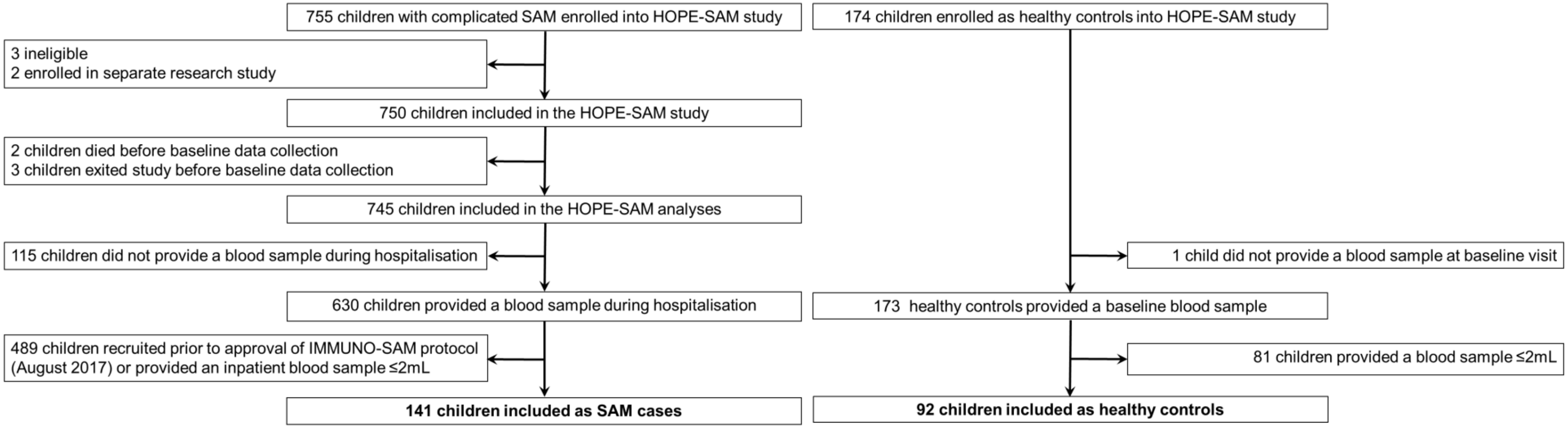

Fig. S1. Selection of children for inclusion in SAM and healthy control groups for analysis of anti-bacterial innate immune cell function.

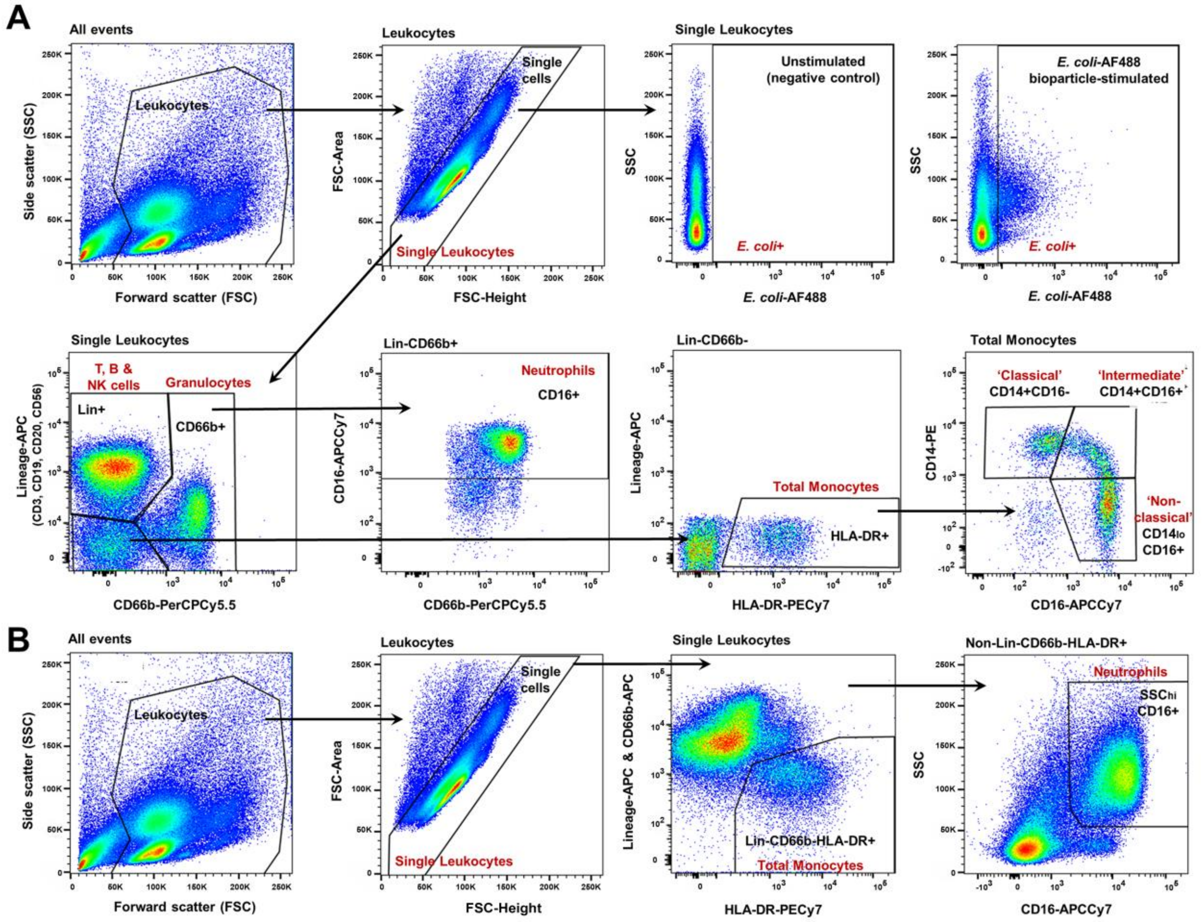

Figure S2. Hierarchical gating strategy for immunophenotyping of cultured blood leukocytes. A) Flow cytometry analysis of cell samples from 1h bacterial binding assays first gated on side-scatter and forward-scatter properties to identify leukocytes and then single cells; sub-populations of single leukocytes were then identified by expression of mixed lymphocyte lineage (Lin) markers (CD3, CD19, CD20, CD56), CD66b, HLA-DR and relative expression of CD14 and CD16. To identify cells that had bound to *E. coli*-coated AF488-labelled bioparticles (*E. coli+*), single leukocytes, leukocyte and monocyte sub-populations were gated on AF488 fluorescence intensity, which was negligible in corresponding negative control assays culture without bioparticles B) Flow cytometry analysis of cell samples from 24h whole blood culture assays first gated on side-scatter and forward-scatter properties to identify leukocytes and then single cells; sub-populations of single leukocytes were then identified by expression of Lin, CD66b, HLA-DR, granularity (side-scatter) and CD16. Activation marker (HLA-DR, CD86, CD40) expression was quantified as median fluorescence intensity within the total monocyte population (Lin-CD66b-HLA-DR+) population. Classical, intermediate and non-classical monocyte sub-sets are not distinguishable after 24h of culture due to reconfiguration of CD14 and CD16 in culture and in response to PAMP.

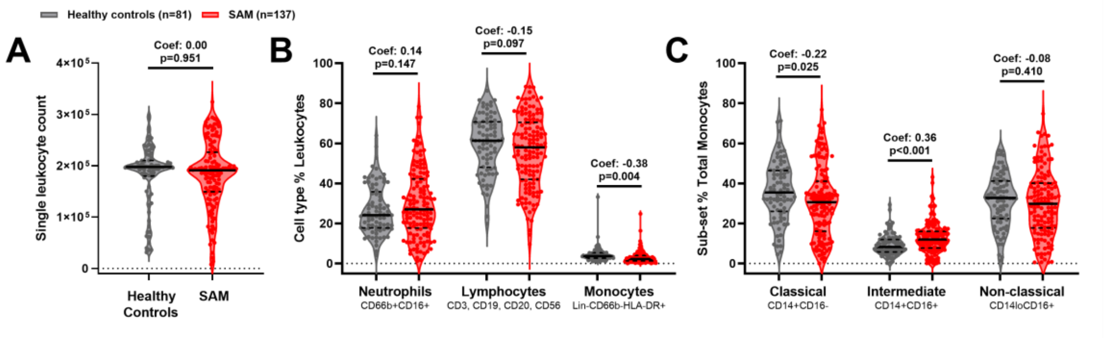

Figure S3. Immune cell types present after 1h culture without bacterial stimulus. Whole blood samples from 137 children with SAM (red) and 81 healthy controls (grey) were incubated for 1h in media alone (unstimulated) as negative controls for the bacterial binding assay or with *E. coli* bioparticles (data shown in **Figure 1**) and analysed by flow cytometry. A) Total single leukocyte counts for flow cytometry analysis, B) leukocyte sub- types as a percentage of total single leukocytes, and, C) monocyte sub-sets as a percentage of total monocytes in unstimulated control cultures. The effect of SAM on immunophenotype was analysed by univariable fractional regression; unadjusted regression coefficients and p-values are indicated.

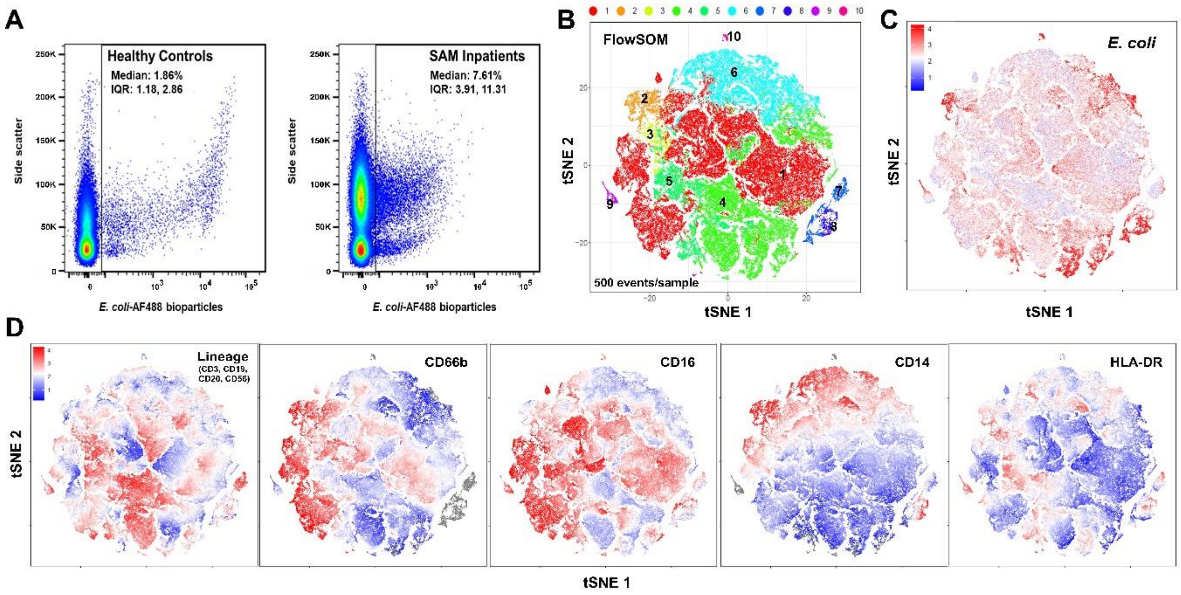

Figure S4. Innate immune cells make up a greater proportion of *E. coli*-binding blood leukocytes in samples from children hospitalised with SAM compared to healthy controls. Whole blood samples from 137 children with SAM and 81 healthy control children were incubated for 1h in media alone (unstimulated) or with 5x10^5 Alexa Fluor 488-conjugated *Escherichia coli* (strain: K-12) bioparticles and analysed by flow cytometry. A) Representative example of flow cytometry gating for all *E. coli*+ single leukocytes for a healthy control (left) and SAM participant (right); median percentage of *E. coli*+ leukocytes and IQR are indicated for each group. B) Annotated FlowSOM analysis (clusters 1-10) of tSNE plot including 500 *E. coli*+ leukocytes from all participants (tSNE separated by healthy control versus SAM group are shown in **Figure 1A**). C) tSNE plot colour-coded for the relative fluorescence intensity of *E. coli*-AF488; all events are *E. coli*+ and AF488 was not included in identification of clusters by FlowSOM. E) tSNE plot colour-coded to indicate intensity of labelling with immunophenotypic antibodies (Lin, CD66b, CD16, CD14, HLA-DR) included in FlowSOM analysis; quantitative analysis of *E. coli*+ proportions and mean AF488 intensities for each cell type are shown in **Figure 1**).

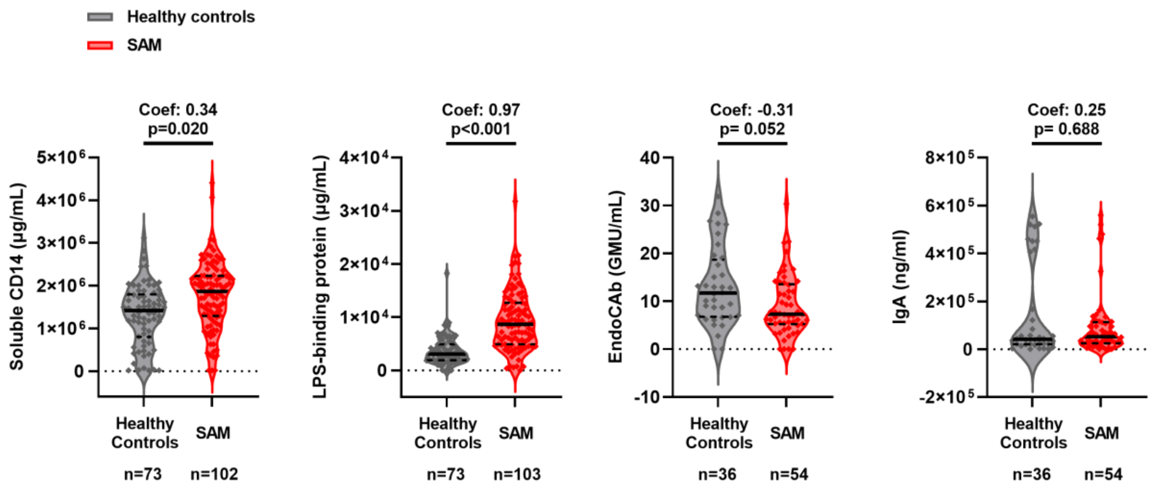

Figure S5. Plasma concentrations of innate and adaptive opsonins in the healthy control and SAM groups. Plasma samples were isolated from the same blood sample used for immune function assays. Innate opsonins (sCD14, LBP) and opsonising antibodies (LPS-specific IgG, total IgA) were quantified via ELISA for participants with sufficient remaining plasma volumes; n indicated for each graph. Unadjusted coefficients (Coef.) and p-values are reported for linear regression of log-transformed concentrations by SAM status.

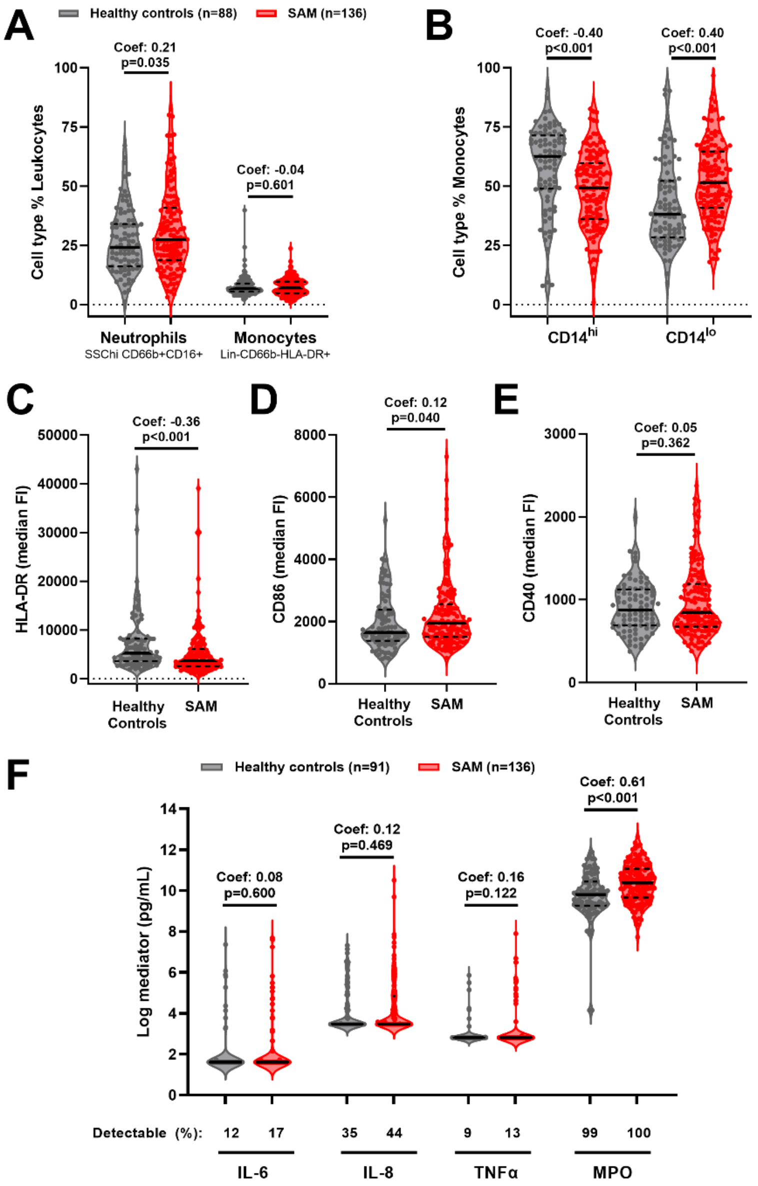

Figure S6. Immune cell types, basal cell surface phenotype and supernatant mediator concentrations present after 24h culture without bacterial stimulus. Whole blood samples from 136 children with SAM (red) and 88 healthy controls (grey) were incubated for 24h in media alone (unstimulated) as negative controls for parallel whole blood cultures with LPS or HKST; cultured cells were immunophenotyped via flow cytometry and soluble mediators in culture supernatants quantified via ELISA. A) Cell types as a percentage of total single leukocytes, and B) monocyte sub-sets as a percentage of total monocytes in unstimulated control cultures. C) Median fluorescence intensity of C) HLA-DR-PECy7, D) CD86-FITC and E) CD40-PerCPCy5.5 on total monocytes. F) Log-transformed concentrations of soluble mediators (pg/mL; censored at the assay-specific limit of detection); percentage of participants in each group with mediator concentrations detectable above the ELISA limit of detection are indicated. The effect of SAM on immunophenotype was analysed by univariable fractional regression (A, B), on monocyte activation/maturation marker expression and mediator concentrations by univariable tobit regression (C-F).

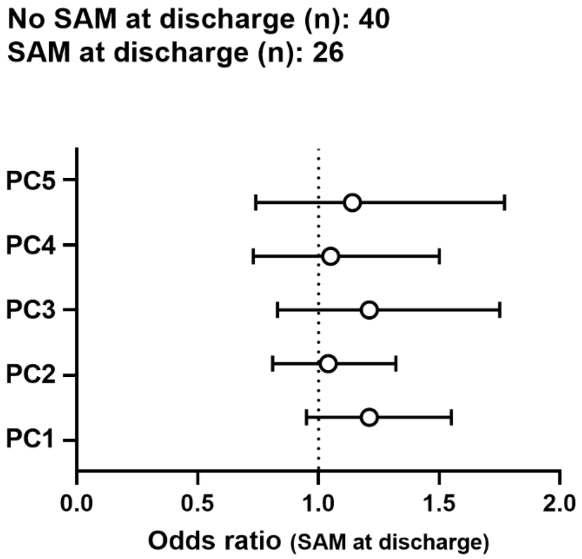

Figure S7. Univariable analysis of anti-bacterial innate immune cell function as predictors of persistent SAM at hospital discharge. Unadjusted odds ratio for persistent SAM at discharge for PC scores among children who had immune function assessment prior to the day of discharge (n=66); adjusted analyses are shown in **Figure 6F**.

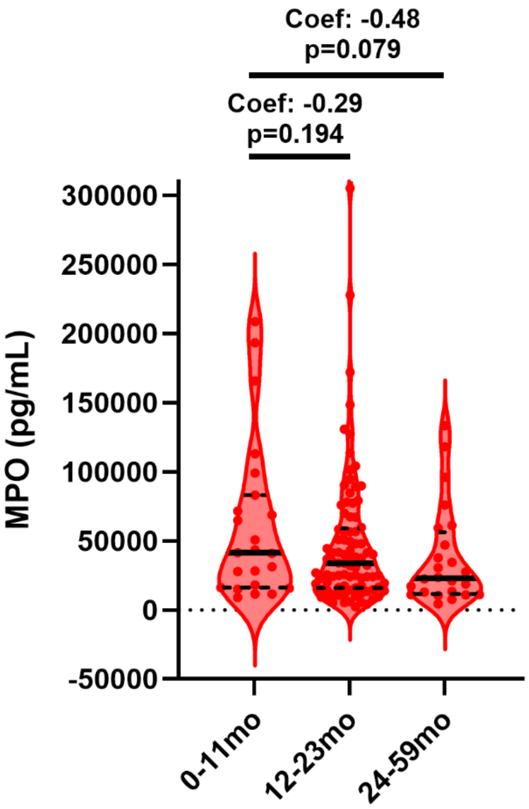

Figure S8. MPO concentrations in unstimulated whole blood culture supernatants from children with SAM by participant age group. Violin plots indicate median (dark line) and IQR (dashed lines); unadjusted coefficients (Coef.) and p-values are reported for univariable tobit regression of MPO by age group for SAM cases (n=136).

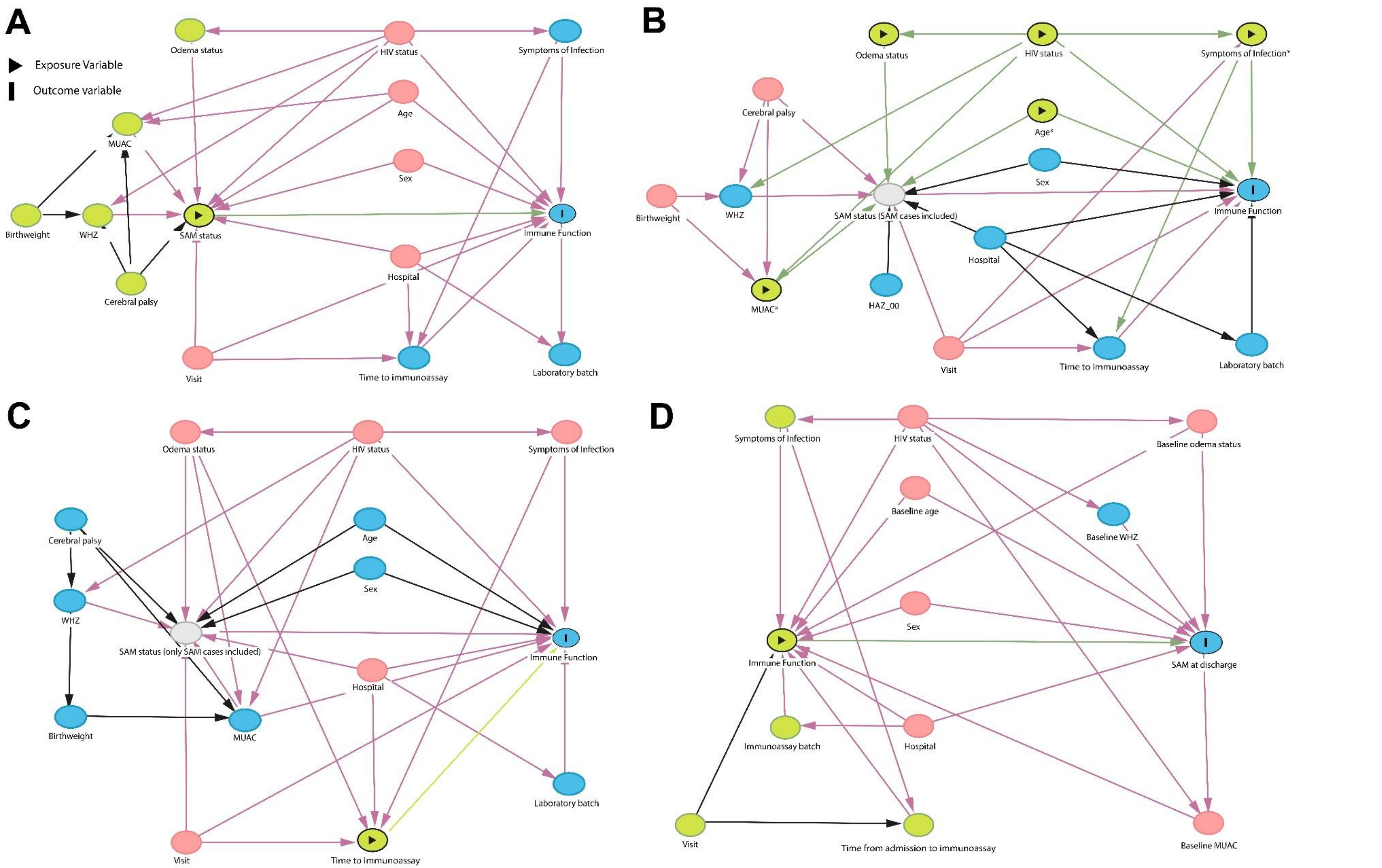
A
Exposure Variable
Outcome variable
Odema status
HIV status
Symptoms of Infection
Age
MUAC
Sex
Birthweight
WHZ
SAM status
Immune Function
Hospital
Cerebral palsy
Visit
Time to immunoassay
Laboratory batch
B
Odema status
HIV status
Symptoms of Infection*
Cerebral palsy
Age*
Sex
Birthweight
WHZ
SAM status (SAM cases included)
Immune Function
Hospital
MUAC*
HAZ_00
Visit
Time to immunoassay
Laboratory batch
C
Odema status
HIV status
Symptoms of Infection
Cerebral palsy
Age
Sex
WHZ
SAM status (only SAM cases included)
Immune Function
Hospital
Birthweight
MUAC
Laboratory batch
Visit
Time to immunoassay
D
Symptoms of Infection
HIV status
Baseline odema status
Baseline age
Baseline WHZ
Sex
Immune Function
SAM at discharge
Immunoassay batch
Hospital
Visit
Time from admission to immunoassay
Baseline MUAC

Figure S9. Directed acyclic graphs (DAG) for hypothesis testing in the IMMUNO-SAM Inpatient cohort. DAGs were constructed using Dagitty software, including all variables with available data from the HOPE-SAM study and for which research literature supported an effect on exposure (I symbol) and/or outcome variables (arrow symbol). Some variables were only measured in children with SAM (e.g. symptoms of infection, time to immunoassay). Green nodes are associated with the exposure only, blue nodes are associated with the outcome only and pink nodes are associated with both the exposure and the outcome (i.e. confounders). A) Causal pathway for the effect of SAM status (exposure) on anti-bacterial immune cell function (outcome), identifying minimal adjustment sets for multivariable models as: sex, age group, HIV infection status, hospital site and visit (i.e. immunoassay on the day of discharge); B) Causal pathway for the effect of clinically-relevant variables (exposures) on anti-bacterial immune cell function (outcome) among children with SAM (healthy controls not included); since there are multiple overlapping causal pathways and limited existing research literature on the effect of these exposures on immune function to support inclusion/exclusion of variables, we used cross-partialling lasso regression with variables of known clinical importance (exposures) fixed in the model and allowed the machine learning algorithm within the model to select variables to control for from the remaining variables to avoid bias; *indicates variables identified in HOPE-SAM data as being associated with post-discharge clinical outcomes. C) Causal pathway for the effect of time to immunoassay (exposure) on anti-bacterial immune cell function (outcome), identifying minimal adjustment sets for multivariable models as: HIV infection status, baseline edema status, hospital site, any symptom of infection at the time of immune function assessment, and visit. D) Causal pathway for the effect of anti-bacterial immune cell function (exposure) on persistent SAM at discharge (outcome) among children with SAM for whom immune function was assessed prior to the day of discharge, identifying minimal adjustment sets for multivariable models as: sex, baseline age group, HIV infection status, baseline edema status, hospital site and baseline MUAC.

SUPPLEMENTARY TABLES

Table S1. Characteristics of children with SAM included in the immune function cohort relative to those in the HOPE-SAM cohort

	IMMUNO-SAM cohort	HOPE-SAM cohort
**N**	**141**	**745**
Hospital site:		
- Harare Central Hospital, n/N (%)	54/141 (38.3%)	278/745 (37.3%)
- Parirenyatwa Hospital, n/N (%)	60/141 (42.6%)	226/745 (32.3%)
- University Teaching Hospital, n/N (%)	27/141 (19.2%)	241/745 (30.3%)
Inpatient mortality, n/N	0/141 (0%)	70/745 (9.4%)
Participant withdrew, n/N	0/141 (0%)	26/745 (3.5%)
Time to discharge, days; median (IQR) [n]	11 (7, 16) [141]	6 (2, 11) [649]
SAM at discharge[1], n/N (%)	58/134 (43.3%)	364/649 (56.1%)
**Participant characteristics (baseline):**		
Age (mo), median (IQR) [n]	19.3 (13.2, 22.3) [141]	17.4 (12.8, 22.1) [745]
- <6 mo	7/141 (5.0%)	20/745 (2.7%)
- 6-11 mo	17/141 (12.1%)	131/745 (17.6%)
- 12-23 mo	92/141 (65.3%)	458/745 (61.5%)
- 24-59 mo	25/141 (17.73%)	136/745 (18.3%)
Male, n/N (%)	71/141 (49.7%)	390/745 (52.4%)
Cerebral palsy, n/N (%)	7/141 (5.0%)	32/745 (4.3%)
**HIV status[1]:**		
HIV positive, n/N (%)	33/141 (23.4%)	162/745 (21.7%)
- On ART, n/N (%)	14/33 (42.4%)	66/162 (40.7%)
**Nutritional status (baseline):**		
Edematous SAM, n/N (%)	98/141 (69.5%)	480/745 (64.4%)
MUAC (mm), mean (SD) [n]	119.7 (18.9) [141]	118 (17.0) [745]
WHZ score, mean (SD) [n]	-2.7 (1.5) [141]	-2.9 (1.8) [745]
WAZ score, mean (SD) [n]	-3.6 (2.0) [141]	-3.8 (1.8) [745]
HAZ score, mean (SD) [n]	-2.9 (1.5) [141]	-3.0 (1.6) [745]

SAM: severe acute malnutrition; IQR: Interquartile range; mo: months; ART: antiretroviral therapy; MUAC: mid-upper arm circumference; WHZ: weight-for-height Z score; WAZ: weight-for-age Z score; HAZ: height-for-age Z score

[1]N for SAM status at discharge (n=134) is due to 8 children in the IMMUNO-SAM cohort being discharged against medical advice (**Table 1**); 1/8 caregivers consented to anthropometry being collected on the day of discharge.

Table S2. Clinical signs and symptoms present in children hospitalized with SAM on the day of their anti-bacterial innate immune function assessment

	SAM Inpatients
**N**	**141**
- Available data on signs/symptoms, n/N (%)	130/141 (92.2%)
Individual signs/symptoms at time of immunoassay[1]:	
Urinary tract infection[3], n/N (%)	0/130 (0%)
Sepsis[3], n/N (%)	1/130 (0.8%)
Meningitis[3], n/N (%)	0/130 (0%)
Malaria[3], n/N (%)	0/130 (0%)
Measles in past 3 months[3], n/N (%)	0/130 (0%)
Tuberculosis (including suspected) [3], n/N (%)	18/130 (13.8%)
Skin infection[3], n/N (%)	0/130 (0%)
Fever, n/N (%)	37/130 (28.5%)
Diarrhea: acute[3], n (%)	34/130 (26.2%)
Diarrhea: persistent[3], n/N (%)	9/130 (6.9%)
Cough[3], n/N (%)	34/130 (26.2%)
Oral thrush[3], n/N (%)	15/130 (11.5%)
Ear Discharge[3], n/N (%)	0/130 (0%)
Cannula site infection[3], n/N (%)	2/130 (1.5%)
Edema, n/N (%)	58/130 (44.6%)
Dermatosis, n/N (%)	15/130 (11.5%)
Shock, n/N (%)	0/130 (0%)
Vomiting, n/N (%)	20/130 (15.4%)
Respiratory distress, n/N (%)	8/130 (6.2%)
Hypothermia, n/N (%)	2/130 (1.5%)
Hypoglycemia, n/N (%)	0/130 (0%)
Dehydration, n/N (%)	3/130 (2.3%)
Lack of appetite, n/N (%)	42/130 (32.3%)
Seizures, n/N (%)	1/130 (0.8%)
Heart Failure, n/N (%)	3/130 (2.3%)
Abnormal electrolytes, n/N (%)	11/130 (8.5%)
Other conditions[2]:	Conjunctivitis[3] (n=1), Angular cheilitis and/or oral sores (n=7), Urticaria (n=1)

[1]Per standardized list of signs/symptoms on daily clinical review form completed on the day nearest the day of immune function assessment. Participants were classified as having ‘any symptom’ on the day of their first immune function assessment based on a study clinician indicating ‘Yes’ for any of the listed symptoms.
[2]Free-text notes but clinician undertaking daily clinical review (i.e. not in standardized list)
[3]Participants were classified as having ‘any symptom/s of infection’ on the day of their first immune function assessment based on a study clinician indicating ‘Yes’ for any of these symptoms, which are typically associated with acute infectious causes. This classification did not include chronic HIV infection or congenital infections associated with non-infectious morbidities in later childhood.

Table S3. Univariable analyses of leukocyte immunophenotype in unstimulated bacterial binding assays and whole blood cultures by SAM status

	Healthy controls	SAM cases	Unadjusted		
**Bacterial binding assays (1h); median (IQR)[1]**	**n=81**	**n=137**	**Coef.**	**95% CI**	**p**
Leukocyte event count[2]	2.0 x10^5 (1.1, 2.2)	1.9 x10^5 (1.5, 2.3)	0.07	-0.04, 0.19	0.220
Lin+ % Leukocytes[3]	61.3 (48.3, 70.5)	58.0 (42.1, 70.3)	-0.15	-0.33, 0.03	0.097
Neutrophils+ % Leukocytes[3]	24.2 (17.9, 35.1)	27.0 (18.0, 42.2)	0.14	-0.05, 0.33	0.147
Monocytes+ % Leukocytes[3]	3.7 (2.7, 5.3)	2.3 (1.3, 4.0)	-0.38	-0.64, -0.12	0.004
- Classical monocytes % Monocytes[3]	35.5 (26.4, 46.4)	30.6 (16.2, 41.0)	-0.22	-0.42, -0.03	0.025
- Intermediate monocytes % Monocytes[3]	8.3 (5.9, 11.9)	12.0 (7.8, 16.0)	0.36	0.18, 0.54	<0.001
- Non-classical monocytes % Monocytes[3]	32.7 (22.7, 41.2)	29.8 (18.1, 40.1)	-0.08	-0.26, 0.11	0.410
**Whole blood cultures (24h); median (IQR)[1]**	**n=88**	**n=136**	**Coef.**	**95% CI**	**p**
Leukocyte event count[2]	1.4 x10^5 (1.1, 1.7)	1.6 x10^5 (1.2, 1.8)	0.13	0.01, 0.25	0.033
Neutrophil % Leukocytes[3]	24.1 (16.2, 34.0)	27.5 (18.8, 40.8)	0.21	0.02, 0.41	0.035
Monocytes+ % Leukocytes[3]	6.8 (5.4, 8.9)	7.1 (4.9, 9.8)	-0.04	-0.21, 0.12	0.601
- CD14hi monocytes % Monocytes[3]	62.6 (49.0, 71.0)	49.3 (36.1, 59.6)	-0.40	-0.59, -0.21	<0.001
- CD14lo monocytes % Monocytes[3]	38.2 (28.6, 52.2)	51.5 (40.9, 64.6)	0.40	0.22, 0.59	<0.001

[1]Untransformed data
[2]Univariable negative binomial regression of untransformed variables
[3]Univariable fractional regression (proportions) of untransformed variables

Table S4. Univariable analyses of innate immune cell bacterial binding capacity by SAM status

Bacterial binding assays (1h)	Healthy controls[1]	SAM cases[1]	Unadjusted[2]		
	n=81	n=137	Coef.	95% CI	p
***E. coli*+ leukocyte count[2]:**					
- Unstimulated assays (negative control)	5.0 (3.0, 8.0)	5.0 (3.0, 10.0)			
- *E. coli* bioparticle-stimulated assays	2,970 (1,967, 5,072)	11,869 (6,842, 21,460)	1.19	0.91, 1.47	<0.001
***E. coli*+ % Leukocytes[3]:**					
- Unstimulated assays (negative control)	0.0 (0.0 ,0.0)	0.0 (0.0 ,0.0)			
- Bioparticle-stimulated assays	1.8 (1.2, 2.8)	7.6 (3.9, 11.3)	1.15	0.88, 1.43	<0.001
**Leukocyte mean FI (*E. coli*-AF488 bioparticles)[4]:**					
- Unstimulated assays (negative control)	0.0 (0.0 ,0.0)	0.0 (0.0 ,0.0)			
- Bioparticle-stimulated assays	74.6 (54.9, 115.0)	23.9 (12.9, 41.1)	-1.16	-0.13, -0.98	<0.001
**Cell types as a percentage of all *E.coli*+ cells[3]:**					
T (CD3+), B (CD19+, CD20+) & NK (CD56+) cells	23.6 (18.0, 36.7)	17.8 (10.3, 27.3)	-0.39	-0.56, -0.21	<0.001
Neutrophils (Lin-CD66b+CD16+)	30.1 (23.1, 43.0)	51.4 (35.7, 63.5)	0.56	0.41, 0.71	<0.001
Monocytes (Lin-CD66b-HLA-DR+)	8.7 (5.6, 12.7)	9.3 (4.3, 15.1)	0.13	-0.11, 0.37	0.277
- Classical monocytes (CD14hiCD16-)	46.2 (29.1, 60.7)	38.2 (23.5, 57.3)	-0.20	-0.37, -0.03	0.021
- Intermediate monocytes (CD14hiCD16+)	7.1 (4.2, 12.3)	8.2 (4.0, 14.2)	0.16	-0.07, 0.39	0.181
- Non-classical monocytes (CD14loCD16+)	14.8 (9.1, 19.5)	14.3 (7.8, 24.0)	0.16	-0.03, 0.34	0.106
***E. coli*+ cells as a percentage of each cell type[3]:**					
T (CD3+), B (CD19+, CD20+) & NK (CD56+) cells	0.8 (0.5, 1.4)	2.1 (1.2, 3.7)	0.90	0.65, 1.15	<0.001
Neutrophils (Lin-CD66b+CD16+)	2.4 (1.3, 3.9)	13.7 (6.9, 21.8)	1.50	1.19, 1.81	<0.001
Monocytes (Lin-CD66b-HLA-DR+)	4.0 (2.7, 6.8)	23.3 (13.2, 38.1)	1.47	1.15, 1.79	<0.001
- Classical monocytes (CD14hiCD16-)	5.0 (2.6, 8.7)	26.1 (11.6, 43.6)	1.15	0.88, 1.43	<0.001
- Intermediate monocytes (CD14hiCD16+)	4.1 (2.5, 8.3)	27.0 (13.5, 41.4)	1.42	1.10, 1.75	<0.001
- Non-classical monocytes (CD14loCD16+)	2.5 (1.4, 4.3)	17.0 (9.1, 30.4)	1.52	1.11, 1.93	<0.001
**Cell type-specific Mean FI (*E. coli*-AF488 bioparticles)[4]:**					
T (CD3+), B (CD19+, CD20+) & NK (CD56+) cells	24.7 (12.6, 38.9)	5.4 (3.0, 11.8)	-1.02	-1.30, -0.74	<0.001
Neutrophils (Lin-CD66b+CD16+)	20.6 (8.8, 45.6)	44.6 (23.3, 68.3)	0.59	0.30, 0.87	<0.001
Monocytes (Lin-CD66b-HLA-DR+)	35.9 (15.6, 130.0)	79.8 (40.3, 143.0)	0.46	0.08, 0.84	0.018
- Classical monocytes (CD14hiCD16-)	25.9 (11.9, 79.1)	74.9 (37.6, 143.0)	0.74	0.38, 1.10	<0.001
- Intermediate monocytes (CD14hiCD16+)	27.0 (8.5, 71.6)	79.7 (34.6, 164.0)	0.89	0.44, 1.35	<0.001
- Non-classical monocytes (CD14loCD16+)	12.0 (5.1, 36.2)	54.0 (22.0, 105.0)	1.20	0.79, 1.62	<0.001

[1]Untransformed median (IQR)
[2]Untransformed values are given (without subtraction); analyses are unadjusted negative binomial regression of log-transformed event counts by SAM status
[3]Untransformed values are given (without subtraction); analyses are univariable fractional regression of log-transformed proportions by SAM status
[4]Untransformed values are given (without subtraction); analyses are unadjusted univariable tobit regression of log-transformed mean fluorescence intensities by SAM status

Table S5. Pairwise correlation of circulating innate and adaptive opsonins with read-outs of bacterial binding capacity from the same children

	Plasma sCD14[1] (µg/mL)		Plasma LBP[2] (µg/mL)		Plasma EndoCAb[3] (GMU/mL)		Plasma IgA[3] (ng/mL)	
	**Coef.**	**p**	**Coef.**	**p**	**Coef.**	**p**	**Coef.**	**p**
***E. coli*+ % Total Leukocytes**	0.21	0.003	0.29	<0.001	-0.14	0.175	-0.07	0.514
**Total Leukocytes *E. coli* meanFI**	-0.07	0.332	-0.275	<0.001	0.24	0.020	0.15	0.147
***E. coli*+ % Lymphocytes**	0.26	<0.001	0.23	0.001	-0.14	0.198	-0.05	0.624
**Lymphocytes *E. coli* meanFI**	0.08	0.272	-0.19	0.008	0.28	0.006	-0.01	0.894
***E. coli*+ % Neutrophils**	0.15	0.034	0.28	<0.001	-0.12	0.270	-0.02	0.883
**Neutrophils *E. coli* meanFI**	0.07	0.362	0.06	0.391	-0.06	0.542	0.15	0.157
***E. coli*+ % Monocytes**	0.20	0.005	0.24	<0.001	-0.18	0.092	-0.12	0.253
**Monocytes *E. coli* meanFI**	0.02	0.794	-0.09	0.240	0.15	0.156	0.00	0.999

GMU – IgG median units

Unadjusted pair-wise correlation coefficients (Coef.) and p-values across both nutritional groups,

[1]Healthy controls n=73, SAM cases n=102

[2]Healthy controls n=73, SAM cases n=103

[3]Healthy controls n=36, SAM cases n=54

Table S6. Univariable analyses of bacterial PAMP-induced monocyte activation markers and mediator secretion by SAM status

Whole blood culture assay (24h)[1]	Healthy controls	SAM cases	Unadjusted		
	n=88	n=136	Coef.	95% CI	p
**Monocyte median fluorescence intensity[2]:**					
**HLA-DR-PE-Cy7**					
- Unstimulated (negative control)	5,252 (3,627, 8,231)	3,706 (2,597, 6,114)			
- LPS-stimulated	15,095 (10,486, 20,027)	8,141 (5,093, 13,577)	-1.29	-1.96, -0.61	<0.001
- HKST-stimulated	11,040 (9,375, 14,819)	7,372 (4,976, 11,725)	-0.38	-1.16, 0.41	0.342
**CD86-FITC**					
- Unstimulated (negative control)	1,651 (1,381, 2,373)	1,948 (1,505, 2,544)			
- LPS-stimulated	2,290 (2,033, 2,843)	2,185 (1,652, 2,818)	-1.39	-2.41, -0.37	0.008
- HKST-stimulated	2,083 (1,820, 2,591)	1,976 (1,639, 2,480)	-1.08	-2.25, 0.10	0.072
**CD40-PerCPCy5.5**					
- Unstimulated (negative control)	874 (690, 1,116)	846 (675, 1,188)			
- LPS-stimulated	1,572 (1,172, 1,952)	1,170 (865, 1,671)	-1.60	-2.34, -0.86	<0.001
- HKST-stimulated	2,124 (1,550, 2,739)	1,481 (1,044, 1,920)	-1.37	-1.88, -0.86	<0.001
**Culture supernatant mediators (pg/mL)[3]:**	**n=91**	**n=136**			
**IL-6**					
- Unstimulated (negative control)	4.0 (4.0, 4.0)	4.0 (4.0, 4.0)			
- LPS-stimulated	258.9 (34.8, 621.6)	65.9 (4.0, 504.1)	-1.48	-2.53, -0.43	0.006
- HKST-stimulated	2,395 (1,286, 4,601)	1,278 (508, 3,386)	-0.60	-1.01, -0.19	0.004
**IL-8**					
- Unstimulated (negative control)	31.2	31.2			

	(31.2, 86.8)	(31.2, 122.2)			
- LPS-stimulated	1,281 (277, 2,971)	543.2 (91.2, 2,366.8)	-1.59	-2.52, -0.67	0.001
- HKST-stimulated	32,062 (13,310, 55,378)	8,875 (2,450, 21,007)	-1.43	-1.89, -0.99	<0.001
**TNFα**					
- Unstimulated (negative control)	15.6 (15.6, 15.6)	15.6 (15.6, 15.6)			
- LPS-stimulated	186.7 (59.5, 459.8)	81.4 (19.9, 268.9)	-1.47	-2.17, -0.77	<0.001
- HKST-stimulated	1,887 (1,022, 3,170)	634.7 (279.3, 1,483.3)	-1.06	-1.40, -0.72	<0.001
**MPO**					
- Unstimulated (negative control)	18,030 (10,447, 34,172)	32,138 (15,726, 62,883)			
- LPS-stimulated	36,986 (23,246, 56,990)	49,990 (22,889, 89,918)	-2.54	-3.99, -1.08	0.001
- HKST-stimulated	64,465 (47,753, 91,344)	70,465 (42,179, 104,769)	-1.59	-2.58, -0.61	0.002

[1]Untransformed median (IQR); values are censored at the assay limit of detection.
[2]Untransformed values are given (without subtraction); analyses are univariable tobit regression of log-transformed differences (Δ) in median fluorescence intensities between antigen-stimulated and unstimulated whole blood cultures (Δ censored at zero, i.e. no PAMP-specific up-regulation) by SAM status
[3]Untransformed values are given (without subtraction); analyses are univariable tobit regression of log-transformed differences (Δ) in supernatant mediator concentrations between monocytes (Lin-CD66b-HLA-DR+) from antigen-stimulated and unstimulated whole blood cultures (Δ censored at zero, i.e. no PAMP-specific up-regulation) by SAM status

Table S7. Univariable and multivariable linear regression of anti-bacterial innate immune function profiles by SAM status

	Unadjusted			Adjusted		
	**Coef.**	**95% CI**	**p**	**Adj. Coef.**[1]	**95% CI**	**p**
**PC1**	3.69	3.03, 4.35	<0.001	3.69	2.97, 4.40	<0.001
**PC2**	-0.48	-1.01, 0.05	0.076	-0.48	-1.13, 0.16	0.143
**PC3**	-0.10	-0.52, 0.32	0.643	-0.23	-0.73, 0.27	0.358
**PC4**	-0.18	-0.49, 0.14	0.278	-0.01	-0.39, 0.38	0.972
**PC5**	0.20	-0.09, 0.48	0.175	0.33	0.03, 0.63	0.031

[1]Healthy controls n=73; SAM inpatients n=126. Model adjusted for sex, age group, HIV infection status, hospital and immune assessment on the day of discharge; confounders for model adjustment selected based on directed acyclic graph (**Figure S9A**)

Table S8. Sensitivity analysis: multivariable linear regression of anti-bacterial innate immune function profiles by SAM status

	HIV-negative only[1]			Prior to discharge only[2]			Day of discharge only[3]		
	**Adj. Coef.[1]**	**95% CI**	**p**	**Adj. Coef.[2]**	**95% CI**	**p**	**Adj. Coef.[3]**	**95% CI**	**p**
**PC1**	4.04	3.17, 4.91	<0.001	3.80	3.09, 4.52	<0.001	3.24	2.34, 4.14	<0.001
**PC2**	-0.33	-1.12, 0.45	0.406	-0.48	-1.14, 0.17	0.146	-0.84	-1.73, 0.05	0.064
**PC3**	-0.22	-0.84, 0.39	0.472	-0.24	-0.76, 0.27	0.350	0.09	-0.50, 0.67	0.769
**PC4**	-0.10	-0.55, 0.35	0.654	-0.01	-0.40, 0.38	0.972	-0.21	-0.66, 0.24	0.361
**PC5**	0.41	0.07, 0.76	0.017	0.36	0.05, 0.66	0.021	0.47	0.03, 0.90	0.036

[1]Healthy controls n=46; SAM inpatients n=101; data plotted in **Figure 4A**. Adjusted for sex, age group, hospital site and assessment on day of discharge.
[2]Healthy controls n=73; SAM inpatients n=75; data plotted in **Figure 4B**. Adjusted for sex, age group, hospital site and HIV infection status.
[3]Healthy controls n=73; SAM inpatients n=51; data plotted in **Figure 4C.** Adjusted for sex, age group, hospital site and HIV infection status.

Table S9. Cross-fit partialling-out lasso regression of PC3 by demographic and clinical variables associated with inpatient and post-discharge mortality, including HEU status

		PC3[1, 2]
**Baseline age group:**		
- **0-11mo**		
	Adj. Coef.	0.43
- **12-23mo**	95%CI	-0.42, 1.28
	p	0.325
	Adj. Coef	0.46
- **24-59mo**	95%CI	-0.53, 1.45
	p	0.364
**HIV status[3]:**		
- **HUU**		
	Adj. Coef.	-0.10
- **HEU**	95%CI	-0.93, 0.73
	p	0.817
	Adj. Coef.	-0.65
- **HIV+**	95%CI	-1.27, -0.03
	p	0.041
**Baseline edema status:**		
- **Non-edematous**		
	Adj. Coef.	0.14
- **Edematous**	95%CI	-0.50, 0.77
	p	0.656
**Symptom/s of infection at time of immunoassay:**		
- **None**		
	Adj. Coef.	-0.07
- **≥1 symptom**	95%CI	-0.61, 0.46
	p	0.795
	Adj. Coef.	0.00
**Baseline MUAC (mm)**	95%CI	-0.02, 0.02
	p	0.787

[1]Controls offered to model: sex, hospital site, time to immunoassay, immune function assessment on the day of hospital discharge, baseline WHZ, baseline HAZ, birthweight & cerebral palsy status; selected based on directed acyclic graph (**Figure S9B**).
[2]3 controls, Wald chi^2: 7.13, p=0.415

Table S10. Univariable and multivariable linear regression of anti-bacterial innate immune function profiles by time between hospital admission and immune function assessment

	Unadjusted			Adjusted		
	**Coef[1].**	**95% CI**	**p**	**Adj. Coef[2,3].**	**95% CI**	**p**
**PC1**	-0.06	-0.12, -0.01	0.017	-0.10	-0.15, -0.05	<0.001
**PC2**	-0.02	-0.06, 0.03	0.482	-0.01	-0.06, 0.05	0.833
**PC3**	0.01	-0.02, 0.04	0.432	0.00	-0.04, 0.04	0.969
**PC4**	0.01	-0.02, 0.05	0.421	0.02	-0.02, 0.06	0.232
**PC5**	-0.03	-0.05, 0.00	0.027	-0.03	-0.05, 0.00	0.043

[1]n=126 children with SAM
[2]n=120 children with SAM; 6 missing data on symptoms of infection
[3]Adjusted for visit, hospital, HIV infection status, baseline edema & symptoms of infection confounders for model adjustment selected based on directed acyclic graph (**Figure S9C**).

Table S11. Summary demographic and clinical characteristics of children with persistent SAM at discharge from hospital

	No SAM at discharge	Persistent SAM at discharge
**N**	**40**	**26**
Hospital site[1]:		
- Harare Central Hospital, n/N (%)	15/40 (37.5%)	14/26 (53.9%)
- Parirenyatwa Hospital, n/N (%)	18/40 (45.0%)	9/26 (34.6%)
- University Teaching Hospital, n/N (%)	7/40 (17.5%)	3/26 (11.5%)
Time to discharge, days; median (IQR) [n]	10.0 (7, 14) [40]	14 (9, 21) [26]
**Participant characteristics (baseline):**		
Age (mo), median (IQR) [n]	19.5 (16, 22) [40]	17.2 (11, 23) [26]
Male, n/N (%)	22/40 (55.0%)	13/26 (50.0%)
Cerebral palsy, n/N (%)	0/40 (0.0%)	3/26 (11.5%)
Birthweight (kg); mean (SD) [n]	3.0 (0.6) [36]	2.9 (0.4) [23]
HIV positive, n/N (%)	8/40 (20.0%)	8/26 (30.8%)
- On ART at admission, n/N (%)	3/8 (37.5%)	1/8 (12.5%)
- On CTX at admission, n/N (%)	4/8 (50.0%)	5/8 (62.5%)
HIV-exposed uninfected, n/N (%)	5/40 (12.5%)	6/26 (23.1%)
HIV-unexposed uninfected, n/N (%)	27/40 (67.5%)	12/26 (46.2%)
**Nutritional status (baseline):**		
Edematous SAM, n/N (%)	31/40 (77.5%)	16/26 (61.5%)
MUAC (mm), mean (SD) [n]	131.2 (15.8) [40]	107.5 (14.5) [26]
WHZ score, mean (SD) [n]	-1.8 (1.7) [40]	-3.6 (2.40) [26]
WAZ score, mean (SD) [n]	-2.6 (1.7) [40]	-4.7 (2.0) [26]
HAZ score, mean (SD) [n]	-2.5 (1.3) [40]	-3.5 (1.7) [26]
- Stunting status (HAZ<-2), n/N (%)	10/40 (25.0%)	19/26 (73.1%)
**Inpatient anti-bacterial innate immune function (pre-discharge):**		
Time to first immunoassay, days; median (IQR) [n]	4.0 (3, 6) [40]	4.0 (3, 11) [26]
PC1 scores, mean (SD) [n]	1.2 (2.4) [40]	2.1 (1.9) [26]
PC2 scores, mean (SD) [n]	0.0 (2.0) [40]	0.2 (2.4) [26]
PC3 scores, mean (SD) [n]	-0.4 (1.4) [40]	0.0 (1.5) [26]
PC4 scores, mean (SD) [n]	-0.2 (1.4) [40]	-0.1 (1.5) [26]
PC5 scores, mean (SD) [n]	0.0 (1.2) [40]	0.2 (1.3) [26]
**Clinical signs & symptoms (day of immunoassay):**		
Any symptom[3], n/N (%)	36/40 (90.0%)	22/26 (84.6%)
Any symptom associated with infection[4]; n/N (%)	24/40 (60.0%)	18/26 (69.2%)
**Nutritional status (discharge):**		
Time from immunoassay to discharge (days), median (IQR) [n]	6.0 (3, 8) [40]	6.5 (2, 13) [26]
Adequate nutrition, n/N (%)	27/40 (67.5%)	-
Moderate acute malnutrition, n/N (%)	13/40 (32.5%)	-
Edematous SAM, n/N (%)	0/40 (0.0%)	1/26 (3.9%)
MUAC (mm), mean (SD) [n]	133.1 (10.7) [40]	108.0 (14.6) [26]
WHZ score, mean (SD) [n]	-1.2 (1.1) [40]	-3.4 (1.5) [26]
WAZ score, mean (SD) [n]	-2.3 (1.4) [40]	-4.8 (1.9) [26]
HAZ score, mean (SD) [n]	-2.7 (1.3) [40]	-3.7 (1.6) [26]
- Stunting status (HAZ<-2), n/N (%)	29/40 (72.5%)	22/26 (84.6%)

Table S12. Univariable and multivariable logistic regression of SAM status at discharge by anti-bacterial innate immune function profiles for children for whom immune function was assessed prior to the day of discharge

	Unadjusted			Adjusted		
	**OR[1, 2]**	**95% CI**	**p**	**Adj. OR[1,3]**	**95% CI**	**p**
**PC1**	1.21	0.95, 1.55	0.120	1.41	1.02, 1.94	0.039
**PC2**	1.04	0.81, 1.32	0.778	0.99	0.68, 1.45	0.971
**PC3**	1.21	0.83, 1.75	0.321	1.56	1.04, 2.35	0.032
**PC4**	1.05	0.73, 1.50	0.796	0.96	0.61, 1.51	0.859
**PC5**	1.14	0.74, 1.77	0.544	0.90	0.41, 2.00	0.790

[1]n=26 children with persistent SAM at discharge, n=40 children with moderate acute malnutrition (-3<WHZ<-2) or adequate nutrition (-2<WHZ) at discharge
[2]Of the original 30 children with persistent SAM and 48 children without SAM at discharge, 8 children (4 in each category) with immunoassay prior to day of discharge were excluded due to discharge against medical advice.
[3]Adjusted for sex, age group. hospital, HIV infection status, baseline edema & baseline MUAC; confounders selected based on directed acyclic graph (**Figure S9D**)

Table S13. **Details of key resources for laboratory methods and data analysis**

REAGENT or RESOURCE	SOURCE	IDENTIFIER
**Cell culture reagents**		
RPMI 1640, Glutamax, +25mM HEPES media	Gibco/Thermo Fisher Scientific	Cat# 72400-021
Penicillin-Streptomycin (10,000 U/mL)	Gibco/Thermo Fisher Scientific	Cat# 15140122
BD FACS Lysing Solution (10x concentrate)	BD Biosciences	Cat# 349202
CO2Gen Compact atmosphere generation sachets	Oxoid	Cat# CD0020
**Bacterial PAMP**		
Alexa Fluor 488-conjugated *E. coli* (K-12 strain) bioparticles	Life Technologies	Cat# E13231
E. coli Lipopolysaccharide (LPS)	Invivogen	Cat# tlrl-3pelps
Heat Killed *Salmonella typhimurium* (HKST)	Invivogen	Cat# tlrl-hkst
**Flow cytometry antibodies**		
APC-conjugated anti-human Lineage mix (CD3, CD19, CD20, CD56); clones: UCHT1, HIB19, 2H7, 5.1H11	Biolegend	Cat# 363601
PerCPCy5.5-conjugated anti-human CD66b; clone: G10F5	Biolegend	Cat# 305108
PE-conjugated anti-human CD14; clone: HCD14	Biolegend	Cat# 325606
APC-Cy7-conjugated anti-human CD16; clone: 3G8	Biolegend	Cat# 302018
PE-Cy7-conjugated anti-human HLA-DR; clone: L243 (G46-6)	BD Biosciences	Cat# 560651
APC-conjugated anti-human CD66b; clone: G10F5	Biolegend	Cat# 305118
FITC-conjugated anti-human CD86; clone: BU63	Biolegend	Cat# 374204
PerCPCy5.5-conjugated anti-human CD40; clone: 5C3	Biolegend	Cat# 334316
Cytometer Setup and Tracking (CS&T) beads	BD Biosciences	Cat# 650622
UltraComp ebeads compensation beads	Invitrogen	Cat# 01-2222-42
**ELISA kits**		
Human TNFα ELISA Duoset	Biotechne	Cat# DY210
Human IL-6 OptEIA Kit	BD Biosciences	Cat# 555220
Human IL-8 ELISA Duoset	Biotechne	Cat# DY208
Human MPO ELISA Duoset	Biotechne	Cat# DY3174
Human CD14 ELISA Quantikine kit	Biotechne	Cat# DC140
Human LBP ELISA Duoset	Biotechne	Cat# DY870
Human EndoCAb IgG ELISA	Hycult Biotech	Cat# HK504-IGG
Human total IgA ELISA	Invitrogen	Cat# 88-50600-22
**Analytical software**		
FACSuite	BD Biosciences	n/a
FlowJo	FlowJo, LLC	Version 10.8.1
Stata	StataCorp, LLC	Version 17.0
R, cytofkit package	R core team	https://github.com/JinmiaoChenLab/cytofkit/; Chen *et al*, PLoS Comput Biol, 2016(66)
